# Lower-Weight-Gain Phenotype Shows Greater Hypothalamic Proteomic Divergence from Control than Higher-Weight-Gain Phenotype in Cafeteria-Diet-Fed Rats

**DOI:** 10.3390/biology15211942

**Published:** 2026-10-26

**Authors:** Sergio Guzmán-Rodríguez, Judith Nwaiwu, Cristian D. Gutierrez-Reyes, Carlos H. López-Lariz, Ricardo Romero-Guevara, Favour Chukwubueze, Oluwatosin Daramola, Tuli Bhattacharjee, Yehia Mechref, Bruno A. Marichal-Cancino

**Affiliations:** 1 Department of Physiology and Pharmacology, Center of Basic Sciences, Autonomous University of Aguascalientes, Aguascalientes 20100, Mexico; sergio.guzman@edu.uaa.mx (S.G.-R.); carlos.lopez@edu.uaa.mx (C.H.L.-L.); ricardo.romero@edu.uaa.mx (R.R.-G.); 2 Department of Chemistry and Biochemistry, Texas Tech University, Lubbock, TX 79409, USA; jnwaiwu@ttu.edu (J.N.); cristian.d.gutierrez-reyes@ttu.edu (C.D.G.-R.); fchukwub@ttu.edu (F.C.); odaramol@ttu.edu (O.D.); tuli.bhattacharjee@ttu.edu (T.B.); yehia.mechref@ttu.edu (Y.M.)

**Keywords:** hypothalamus, weight gain, proteomics, cafeteria diet, behavior

## Abstract

Cafeteria diet, rich in fat and sugar, rapidly induces metabolic dysfunction and alters central energy regulation. We examined behavior and hypothalamic protein abundance profiles in cafeteria-diet-fed rats classified into weight-gain quartiles (Q1–Q4) and compared with standard-chow-fed controls. Pre-adolescent male Sprague–Dawley rats (n = 39:10 controls; Q1–Q4, n = 7–8 each) received ad libitum access to standard chow or a palatable cafeteria diet and underwent open field testing followed by quantitative LC–MS/MS proteomics of the hypothalamus. Behavioral assessment showed no major differences in locomotion, anxiety-like parameters, or exploration, except for reduced grooming (*p* ≤ 0.05) in cafeteria-fed versus control animals. Global, threshold-independent analyses showed that the lower-weight-gain phenotype (Q1) was significantly more distant from controls than the higher-weight-gain phenotype (Q4) (multivariate distance to the control centroid; Mann–Whitney U, *p* = 0.006), a pattern also observed by principal component analysis and hierarchical clustering. Consistently, of 17 proteins that remained significant after false discovery rate correction, 16 differed between Q1 and control and only one between Q4 and control, despite both phenotypes having ad libitum access to the same cafeteria diet. These findings suggest that lower weight gain is associated with more extensive hypothalamic protein abundance changes, although a causal link remains to be established, and they highlight candidate pathways for future mechanistic studies.

## Simple Summary

In this study, rats fed a hypercaloric cafeteria-type diet showed divergent weight gain, allowing classification into quartiles (Q1–Q4) relative to standard-chow-fed controls. Despite being maintained under comparable experimental conditions, cafeteria-fed rats showed only a modest reduction in grooming relative to controls, with no other differences in locomotion or anxiety-like behavior in the open field (OF) paradigm. Nonetheless, global analyses of the hypothalamic proteome (principal component analysis, hierarchical clustering, and the distance of each animal to the control profile) showed that the lowest-weight-gain phenotype (Q1) diverged markedly from controls, whereas the highest-weight-gain phenotype (Q4) remained close to them. Consistently, 16 of the 17 proteins that remained significant after correction for multiple testing differed between Q1 and controls, compared with only one between Q4 and controls.

## 1. Introduction

The global rise in obesity has been largely driven by the widespread availability of calorie-dense, highly palatable foods. In humans, diets rich in processed foods (Western), which are often high in fat and sugar, promote excess weight gain, insulin resistance, and low-grade inflammation [1]. Rodent cafeteria diet models have been developed to mimic this dietary pattern by offering animals a variety of human junk food items (e.g., cookies, processed meats, chips) alongside standard chow. These cafeteria diets induce rapid and severe adiposity and metabolic syndrome, often exceeding that produced by conventional high-fat chow. For example, Sampey et al. found that rats fed a cafeteria regimen for 15 weeks showed dramatically higher body weight, hyperglycemia, and hyperinsulinemia than those on a matched 45% high-fat diet [2]. Cafeteria-fed rodents also exhibit pronounced inflammation in the liver and adipose tissues. In sum, cafeteria feeding provides a robust model of human metabolic syndrome characterized by exaggerated obesity, glucose intolerance, and systemic inflammation [2,3]. These models are therefore widely used to study the effects of obesogenic diets on energy balance.

Notably, cafeteria diets affect not only metabolism but also behavior. Rodents given cafeteria diets typically consume more calories and show altered reward-related behavior (hyperphagia, preference for palatable foods) compared to controls [2,4]. Chronic exposure to such diets has been linked to changes in mood and cognition: for instance, short-term cafeteria feeding induced anxiety-like behavior in adult rats alongside elevated inflammatory cytokines in the brain [5]. Moreover, abrupt withdrawal from a long-term palatable diet can precipitate depressive-like phenotypes, accompanied by dysregulation of dopaminergic and endocannabinoid signaling in brain reward areas [5]. In humans, overconsumption of junk foods and the resulting obesity are correlated with higher rates of anxiety and depression, suggesting that diet-induced neural changes may influence emotional state [6].

Together, these findings underscore that cafeteria diets perturb neural circuits of reward and stress, likely via systemic and central inflammatory mediators [5].

In addition, central to body weight homeostasis is the hypothalamus, which integrates hormonal and nutrient signals to regulate hunger and satiety. Within the hypothalamic arcuate nucleus (ARC), two antagonistic neuron populations orchestrate feeding: orexigenic agouti-related peptide/neuropeptide Y (AgRP/NPY) neurons that stimulate appetite, and anorexigenic proopiomelanocortin (POMC/CART) neurons that suppress it [7]. Under normal conditions, adiposity signals such as leptin (from fat) and insulin (from the pancreas) act on these neurons to limit food intake. This coordinated hormonal control keeps energy intake in balance with stores.

In addition to leptin and insulin, hypothalamic neurons integrate direct nutrient cues via intracellular pathways. The mechanistic target of rapamycin complex 1 (mTORC1) is a key nutrient energy sensor in the hypothalamus. Hypothalamic mTORC1 activity responds to changes in amino acids and cellular energy and is colocalized with AgRP/NPY and POMC neurons [8]. Thus, mTORC1 acts as a cellular fuel gauge whose hypothalamic activity regulates feeding in accordance with nutrient availability [8]. In this way, in obesity, these appetite regulatory pathways become disrupted; high-fat or cafeteria diets induce central leptin and insulin resistance, weakening the usual satiety feedback [7,9]. In summary, these molecular pathways normally restrain eating but are dysregulated by obesogenic diets, leading to appetite imbalance.

On the other hand, a growing body of evidence indicates hypothalamic neuroinflammation as a driver of obesity and metabolic disturbance. For instance, in rodent models, consumption of high-fat or cafeteria diets rapidly activates inflammatory cascades in the hypothalamus, even before significant weight gain [9]. Indeed, blocking key inflammatory mediators (e.g., IKKβ/NF-κB) in hypothalamic neurons protects against diet-induced leptin resistance and weight gain, confirming a causal role for central inflammation in obesity pathogenesis.

Neuroinflammatory changes with obesity are not limited to rodents. In humans, imaging studies have detected signs consistent with hypothalamic gliosis in obese subjects [9]. Dorfman and Thaler likewise reported that obesity is associated with microglial activation and loss of hypothalamic neuronal projections in humans [10]. These central inflammatory responses correlate with peripheral insulin resistance, suggesting crosstalk between brain and metabolic disease. Moreover, individual susceptibility to obesity in animal models often correlates with the magnitude of hypothalamic inflammation; obesity-prone rats exhibit stronger inflammatory gene induction and gliosis on cafeteria feeding than obesity-resistant rats [10].

In the context of cafeteria feeding, these inflammatory processes may interact with behavioral changes. For example, short-term cafeteria diets not only increase adiposity but also elevate proinflammatory cytokines (e.g., TNF-α) in the amygdala and plasma, which is accompanied by anxiety-like behavior [5].

Proteomic studies are opening new venues for addressing the multifactorial nature of pathologies such as obesity. In this regard, recent proteomic studies have begun to elucidate the molecular basis of hypothalamic dysfunction in obesity. For example, Xi et al. [11] performed a comparative proteomic analysis of the hypothalamus in diet-induced obese (DIO) rats versus diet-resistant (DR) ones. Significantly, DR rats showed higher levels of proteins (e.g., CaMKV, BASP1) related to synaptic plasticity and lower markers of inflammation than DIO rats [11]. Similarly, our group recently carried out a functional enrichment analysis using proteomic data, which highlighted differences in cytoskeleton organization, neurite formation, and synaptic vesicle cycling when comparing higher- vs. lower-weight-gain rats [12]. However, whether these proteomic changes are a cause or effect of obesity is currently unknown, and more mechanistic studies are needed before we can conclusively identify hypothalamic features associated with lower weight gain. Nonetheless, so far these findings suggest that diet-resistant animals [11] maintain a hypothalamic proteome with increased neuronal activity and reduced inflammation. In contrast, rats with higher weight gain exhibit proteomic profiles consistent with dysregulated aerobic metabolism as recently reported [13]. In this study, we build on our previous work by analyzing the hypothalamic proteome of a standard-chow control group that was studied simultaneously with the cafeteria-diet-fed rats but not included in our previous analysis, and comparing it with that of the cafeteria-diet-fed rats stratified a posteriori into weight-gain quartiles (Q1–Q4). The present analysis therefore focuses on comparing each cafeteria-diet quartile with the standard-diet control group and, with the aim of identifying significantly different expressed proteins (adjusted Tukey *p* ≤ 0.05) potentially associated with the lower-weight-gain phenotype. Notably, global analyses of the hypothalamic proteome showed that the lower-weight-gain phenotype (Q1) diverged more from control than the higher-weight-gain phenotype (Q4), a pattern also supported by FDR-corrected protein-level analysis. This suggests that hypothalamic protein abundance differences cannot be explained entirely by diet or weight gain alone; whether they reflect pre-existing differences between groups requires direct testing. The relationship between the present dataset and the previously reported Q1-vs-Q4 comparison [12], including the extent of overlap in biological samples and/or proteomic datasets, is explicitly described below.

## 2. Methods

### 2.1. General Methods

**Figure 1:**
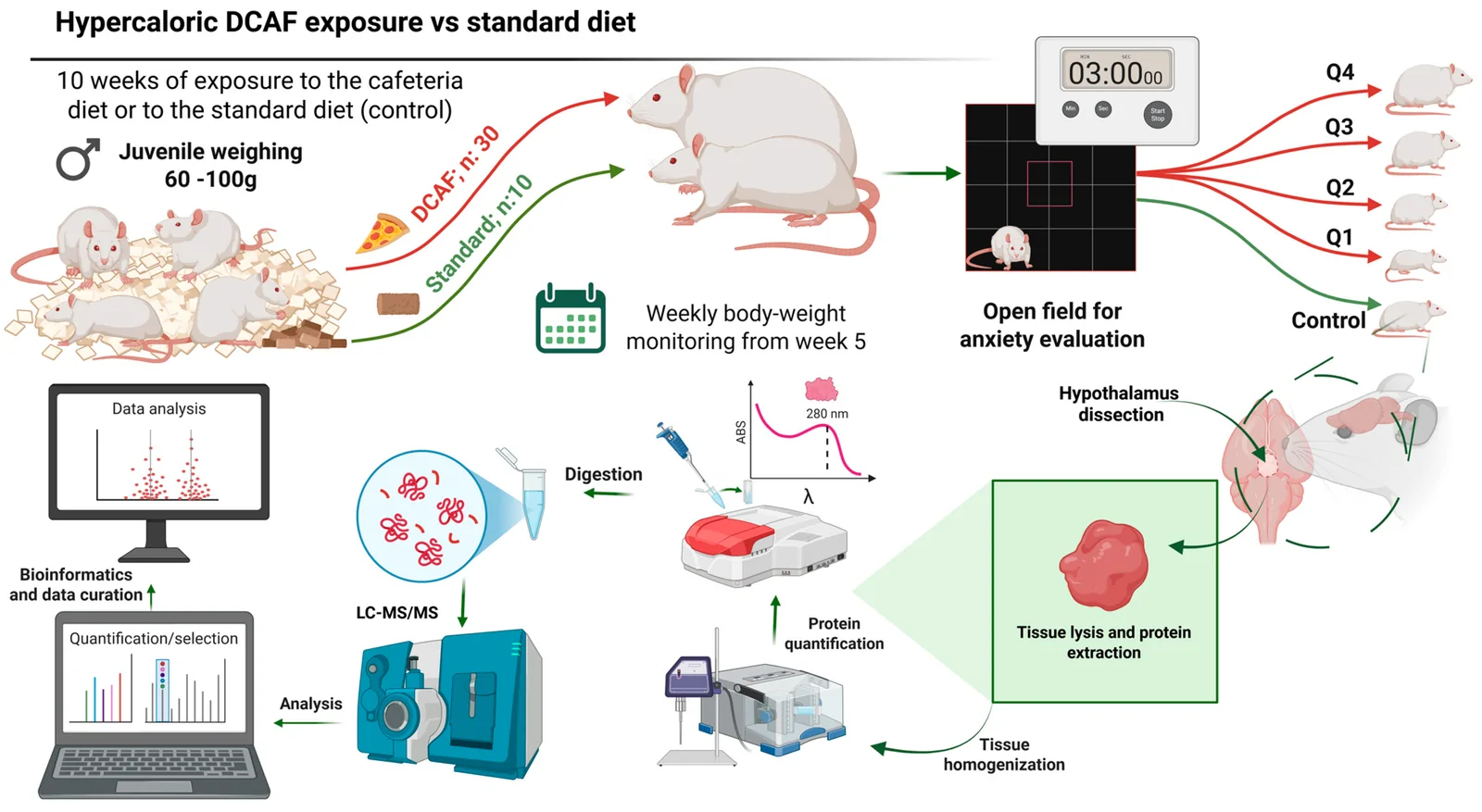
Diagram of experimental protocols. The protocol shows 30 rats subjected to a cafeteria diet for a period of 10 weeks compared to a control group of 10 rats subjected only to a standard diet, pair-age-matched. Subsequently, open field (OF) behavioral tests and hypothalamic proteomics were performed. Created in BioRender. Marichal, B. (2026) https://BioRender.com/ayxqfu5.

The previously published comparison [12] was limited to the Q1 vs. Q4 quartiles of the cafeteria-diet-fed animals only. For the present study, we included a control animal cohort for both the proteomic and behavioral analyses (Figure 1). Consequently, every analysis presented here that involves the control group, including the quartile-vs-control comparisons, is entirely original. Furthermore, the animals from both groups (the control group and the group fed a cafeteria diet) were analyzed simultaneously. This means they were studied during the same timeframe, albeit with different research objectives. The only previously generated data reused in this manuscript is the Q1-vs-Q4 differential protein list from [12], used exclusively for the exploratory set and functional analysis (see Results). No other previously published figures or results are reproduced. All control animals were housed in a single cage (n = 10), whereas cafeteria-fed animals were randomly distributed across five cages (5–6 rats each); the implications of this design for the control-versus-quartile comparisons are addressed in the Discussion Section 4.3.

### 2.2. Animals

Forty pre-adolescent male Sprague–Dawley rats between 3.5 and 4 weeks old, weighing 60 to 100 g (at the start of the experiments) and obtained from the Autonomous University of Aguascalientes animal facility, were housed in maintenance cages; there were five cages for the cafeteria-diet groups (in groups of 5 to 6 rats per cage), plus the cage for the control group (10 rats). Animals were randomly allocated to the control or cafeteria-diet condition. Rats were in the habitat of our research vivarium for a week before any manipulation. Prior to any experimental manipulation, the animals underwent a 1-week acclimation period within our institution’s vivarium facility. All rats in this study were maintained under a 12/12 h light/dark cycle (with the light phase starting at 10:00 a.m.) at a controlled temperature of 22 ± 1 °C, and a humidity of 50%. Animals were housed in standard cages with access to standard food or cafeteria diet, and water *ad libitum*. Body mass was recorded weekly throughout the 10-week protocol in the cafeteria-diet groups for our previous publication [12]; in the control group, body mass was recorded at baseline and then weekly in the last 5 experimental weeks, to match the comparison window used in our previous analysis [12]. Midway through the experimental period, one rat developed clinical signs of otitis and was subsequently isolated, treated, and excluded from the study to prevent potential pathogen transmission to the remaining colony. Consequently, the final sample sizes for the experimental cohorts were control n = 10, Q1 n = 7, Q2 n = 8, Q3 n = 7, and Q4 n = 7.

The experimental procedures were conducted in accordance with the guidelines approved by the Institutional Ethics Committee (PIFF24-1 CEADIUAA—approved on 28 June 2022). Furthermore, all protocols were performed in strict compliance with both national and international regulatory frameworks for the care and use of laboratory animals prescribed by the United States of America, as well as the Animal Research: Reporting of In Vivo Experiments (ARRIVE) guidelines [14,15].

### 2.3. Access to Overfeeding

Cafeteria-diet animals had free access to an overfeeding protocol using a cafeteria diet, adapted from previous studies [16]. The nutritional formulation comprised 400 g of sausage, 200 g of Oreo biscuits, 200 g of Maria biscuits, 300 g of lard, 100 g of pancake flour, and 300 g of ground standard rodent chow. The cafeteria diet provided ~35% higher energy density than standard chow (i.e., 4.26 kcal/g vs. 3.15 kcal/g, respectively). Briefly, the ingredients were mixed until a croquette was obtained, and they were wrapped and refrigerated at −5 °C. Based on our previous experiments, the overfeeding period lasted 10 weeks [12]. The control animals used in this study were age-paired to cafeteria diet ones. The initial body weight at the beginning of the 10-week experimental protocol (*ad libitum* access to water and standard feed) was recorded in control animals, as well as their weekly body weight in the last 5 weeks. These weekly recordings (weeks 6–10) were decided based on the statistical differences among quartiles, as observed during that time frame (weeks 6 to 10) in our previous publication [12].

Cafeteria-fed animals were ranked according to their body weight at the end of the 10 weeks of experimentation as follows: the 7 rats with the lowest body weight were categorized as Q1, followed by the next 7–8 (intermediate Q2–Q3), and so on until the last 7 with the highest weight, marked as Q4. Q1–Q4 animals were distributed across all five cafeteria-diet cages (C1: Q1 = 1, Q2 = 3, Q4 = 2; C2: Q3 = 2, Q4 = 3; C3: Q1 = 1, Q2 = 1, Q3 = 2, Q4 = 2; C4: Q1 = 4, Q2 = 1, Q3 = 1; C5: Q1 = 1, Q2 = 3, Q3 = 2) rather than segregated by cage, and a permutation test on this cage-by-quartile distribution found no significant association between cage identity and quartile membership (*p* = 0.089). Because Q1–Q4 were defined using absolute body mass, this variable was used strictly as a stratification criterion; it was not subsequently treated as independent evidence of lower or higher weight gain, which instead relies on the longitudinal weight trajectories and the divergent proteomic and behavioral profiles reported below. This post hoc classification may introduce partial overlap between adjacent quartiles, a limitation addressed in the Section 4.

### 2.4. Open Field Test

Anxiety-like phenotypes were quantified via the open field paradigm, which utilized a square-shaped stand (60 cm per side). This enclosure featured a black background demarcated into two focal areas: a central area (20 cm per side) and the surrounding periphery. Each rat was allowed to explore the apparatus freely for a single 3-min trial. Animal behavior was monitored continuously via video tracking; subsequently, the frequencies of rearing (standing on their hind legs), grooming, and entries into both the central quadrants and periphery were scored manually through blind visual analysis. Additionally, the time spent per quadrant, distance traveled, and freezing behavior were analyzed by Fiji (Image J 2.16.0/1.54p) software version Java 1.8.0_322 [17] and its plug-in Animal Tracker API [18]. Rearing and grooming were scored visually by an observer aware of group assignment, without formal blinding; the remaining parameters (time per quadrant, distance traveled, freezing) were extracted automatically via this software and were therefore less subject to observer bias.

### 2.5. Hypothalamus Extraction

Following euthanasia at the conclusion of the experimental timeline, the hypothalamus was dissected along with other tissue samples (not evaluated in the present study). The hypothalamic region was identified visually, as described in previous literature (for both the control and cafeteria-diet groups, which were simultaneously collected and stored under the same conditions) [19,20], based on the stereotaxic coordinates of AP: −3.36; ML: 2.0; and DV: 8.0, following the anatomical atlas of Paxinos and Watson [21]. Tissue isolation was performed via a sagittal cut followed by the segmentation of the hypothalamus between the optic chiasm and the cerebellum. The isolated hypothalamus, yielding a circular tissue mass of around 0.5 cm in length, was subsequently stored at −80 °C for cryopreservation.

Animals were euthanized by isoflurane (Sofloran^®^, Laboratorios PiSA S.A. de C.V., Guadalajara, Jal., Mexico) exposure to induce deep anesthesia, followed immediately by thoracotomy and brain extraction (to obtain tissue samples) as the secondary physical method to ensure death, in accordance with standard laboratory-animal euthanasia guidelines.

### 2.6. Tissue Lysis and Protein Extraction

The cryopreserved hypothalamus tissues (maintained at −80 °C) were thawed and subsequently lysed within a 2 mL microcentrifuge tube containing approximately 100 mg of 400 µm zirconium beads. Thereafter, a volume of 150 µL of 50 mM ammonium bicarbonate (ABC) buffer and 150 µL of 5% sodium deoxycholate (SDC) were added to the tubes. The samples were homogenized using a Beadbug microtube homogenizer (Benchmark Scientific, Edison, NJ, USA) at 4000 g for 30 s. This homogenization cycle was performed three times with 30 s resting intervals on ice, followed by sonication for 1 h. Afterward, the samples were centrifuged at 14,800 g for 10 min, and the supernatant containing the solubilized proteins was collected and transferred to a new tube. Protein concentrations were quantified using a bicinchoninic acid (BCA) assay kit (Thermo Scientific). Samples from all groups were prepared and analyzed by LC–MS/MS within the same timeframe, with randomized sample processing.

### 2.7. Tryptic Digestion

An aliquot containing 50 µg of protein from each sample was retrieved from the tissue stock and transferred to a sterile 1.5 mL microcentrifuge tube. Thermal denaturation was performed at 90 °C for 15 min. Subsequently, disulfide bonds were reduced by the addition of 1.25 µL of 200 mM dithiothreitol (DTT), followed by incubation at 60 °C for 45 min. Free sulfhydryl groups were then alkylated using 5 µL of iodoacetamide (IAA) under incubation at 37.5 °C for 45 min in the dark. To quench the alkylation reaction, 1.25 µL of DTT was added and incubated at 37.5 °C for 30 min. Enzymatic digestion was executed using trypsin at a 1:25 (enzyme-to-protein) mass ratio under overnight incubation (~18 h) at 37.5 °C. Proteolysis was quenched with 1 µL of formic acid, and the samples were dried using a SpeedVac concentrator. The resulting peptides were subsequently purified via a C18 top-tip desalting protocol and then dried down in the SpeedVac concentrator. Finally, the dried purified peptides were reconstituted in 2% ACN, 98% HPLC-grade water, and 1% formic acid to a final concentration of 1 µg/µl before LC–MS/MS analysis.

### 2.8. Proteomics LC-MS/MS Conditions

For the LC-MS/MS analysis, a 1 µg aliquot of each sample was inserted into a nano UltiMate 3000 Nano UHPLC system (Thermo Sci., San Jose, CA, USA) coupled with an Orbitrap Fusion Lumos mass spectrometer (Thermo Sci., San Jose, CA, USA) operated in positive mode. Mobile phase A (MPA) comprised 2% ACN in HPLC-grade water with 0.1% FA, and mobile phase B (MPB) contained 100% ACN with 0.1% FA. Initially, samples were loaded onto an Acclaim PepMap 100 C18 trapping column (75 μm × 2 cm, 3 μm particle size, 100 Å pore size; Thermo Scientific, Pittsburgh, PA, USA). Chromatographic separation was then performed on a reversed-phase C18 Acclaim PepMap column (15 cm × 75 μm; Thermo Scientific, Pittsburgh, PA, USA), keeping the column temperature constant at 29.5 °C throughout the 120-min analysis run time. The applied multistep gradient began at 5% MPB for 10 min, followed by 30% over 75 min, 50% over 13 min, and 90% over 2 min, which was kept constant at 90% for 4 min to wash the column. Finally, MPB was decreased to 5% in 1 min and held constant for 4 min to equilibrate the column. The eluting peptides were inspected in positive mode on the Orbitrap mass analyzer. The full MS spectra were acquired by the Orbitrap mass analyzer with a resolving power of 120 K, covering a mass-to-charge (*m*/*z*) range of 350 to 1800. The AGC target value was set to 1 × 10^6^, the maximum injection time was set to auto, and the RF lens was maintained at 30%. The dynamic exclusion parameters were set as follows: repeat count of 1; exclusion duration of 60 s; and a mass tolerance of 10 ppm. The MS2 scans were produced in a data-dependent acquisition (DDA) mode, where the top 20 most intense precursor ions were selected for fragmentation via stepped higher-energy collision dissociation (HCD) at 15%, 25%, and 35%. The isolation mode utilized was the quadrupole with an isolation window of 1.6 *m*/*z*. The AGC target value was set to the standard of 1 × 10^6^, the maximum injection time was set to auto, charge states 2–8 were included, and the normalized AGC target was maintained at 100%.

### 2.9. Proteomic Data Analyses

Proteomic raw data analysis was executed utilizing Proteome Discoverer 2.5 software (version 2.50.400). The UniProt Swiss-Prot rat FASTA database (downloaded on 20 March 2025) served as the reference for the database search. Fixed modifications were defined as the carbamidomethylation of cysteine, whereas methionine oxidation and protein N-terminal acetylation were incorporated as variable modifications. Downstream analysis was restricted exclusively to proteins with more than one peptide. The mass tolerances for precursors and fragments were established at 10 ppm and 0.02 Da, respectively. Peptide and protein identifications were filtered at a 1% false discovery rate. Protein abundances were then relatively normalized using the total sum of identified proteins in each sample as 100 (%). Normalized relative abundance data were then used to identify significantly different expressed proteins across groups (ANOVA *p* ≤ 0.05) and FDR-adjusted ANOVA. For multiple comparisons (e.g., Control vs. Q1, Control vs. Q2, etc.; α = 0.05), a Tukey post hoc test was performed to control the family-wise error rate; the resultant significantly different expressed proteins between pair comparisons (Adjusted *p* ≤ 0.05) were then used for exploratory set analysis (Venn diagrams) and functional enrichment analysis [22,23]. Proteomic samples were shipped to and processed by the collaborating laboratory (Texas Tech University) blind to group/quartile identity. Keratins, which may partly reflect exogenous contamination during sample handling, were retained in the global dataset; excluding them (21 of 1294 proteins) did not change the multivariate results (Q1 vs. Q4 distance to control centroid, *p* = 0.009). Keratin 1, the only keratin reaching FDR significance, was excluded from the FDR-supported protein set as a probable contaminant.

Given the exploratory nature of this study and its emphasis on identifying coordinated biological processes rather than establishing individual proteins as diagnostic or prognostic biomarkers, differential protein abundance data (Tukey-adjusted *p* ≤ 0.05) were used for functional analysis, since the FDR correction drastically decreased the number of identified proteins, limiting their use in functional analysis. Accordingly, results are reported at two levels: FDR-supported proteins and exploratory candidate proteins identified by Tukey-adjusted pairwise comparisons, the latter used only for hypothesis generation. In this modest-sized cohort (n = 7–10 per group), protein-level FDR correction would substantially reduce sensitivity and could exclude coordinated but moderate changes that remain biologically informative at the pathway level. Therefore, proteins meeting the significance criterion were treated as candidates for hypothesis generation and were used to investigate broader functional patterns through set analysis and pathway-enrichment analyses. No individual protein was considered independently validated or interpreted as a definitive biomarker based solely on these results. Biological conclusions were based on convergent pathway-level evidence, together with the threshold-independent multivariate analysis described below, rather than on the significance of individual proteins, which serve as hypothesis-generating elements.

Mean relative abundance data were transformed into raw Z-scores (proteins), and then used for hierarchical clustering analysis, with correlation distance and average linkage as the clustering algorithm. The heatmap was generated in the “ClustVis” web-based tool [24]. To complement this group-level clustering with another quantitative, sample-level analysis, individual protein relative abundance values for each of the 39 biological replicates were log2-transformed and standardized to raw Z-scores per protein. One control sample (Control_8) was excluded from this analysis based on a pre-specified quality criterion: its missing value rate (2.8% of the 1294 proteins) was an order of magnitude higher than any other sample (≤0.15%), consistent with a technical acquisition issue; including it did not change the direction or significance of the result. Proteins with any missing value across the remaining 38 samples were excluded (1282 of 1294 retained). Principal component analysis (PCA) of the group-mean profiles (control and Q1–Q4) was performed to visualize the global proteomic structure. For each sample, the Euclidean distance to the control group centroid was calculated in this standardized protein space (leave-one-out centroid for control samples), and distances were compared between the Q1 and Q4 phenotypes using the Mann–Whitney U test. This analysis was performed with the assistance of Claude Sonnet 5 (Anthropic).

We executed a functional enrichment examination using the online tool “DAVID” [25,26] and the software “Cytoscape,” version 3.10.4, and its built-in “Clue Go app” version 2.5.10 [22,23]. This analysis sought to reveal which functional categories, including biological processes, molecular functions, cellular components, and signaling or metabolic pathways, were overrepresented among the significantly different expressed proteins we detected. Only significantly different expressed proteins between control (standard chow) and quartiles Q1 to Q4 were used for the analysis (Tukey-adjusted *p* ≤ 0.05). The reference terms for enrichment analysis were derived from the Gene Ontology Consortium database (GO: biological process, molecular function, immune system, and cell compartment), as well as the KEGG, Reactome, and Wikipathways databases. The setup for network generation established biological terms at levels 3–12, and only terms enriched with *p* ≤ 0.05 were included in our analysis. We used a term fusion layout in the ClueGo setup, as well as a kappa score of 0.5 for the network connectivity algorithm.

### 2.10. Data and Statistics

Behavioral metrics were subjected to Student’s *t*-test, paired Kolmogorov–Smirnov test, and Wilcoxon test. Proteomic data acquisition and analysis are described in the previous section. In both cases, statistical significance was defined as *p* ≤ 0.05.

## 3. Results

### 3.1. Effects of the Cafeteria Diet on Body Mass

**Figure 2:**
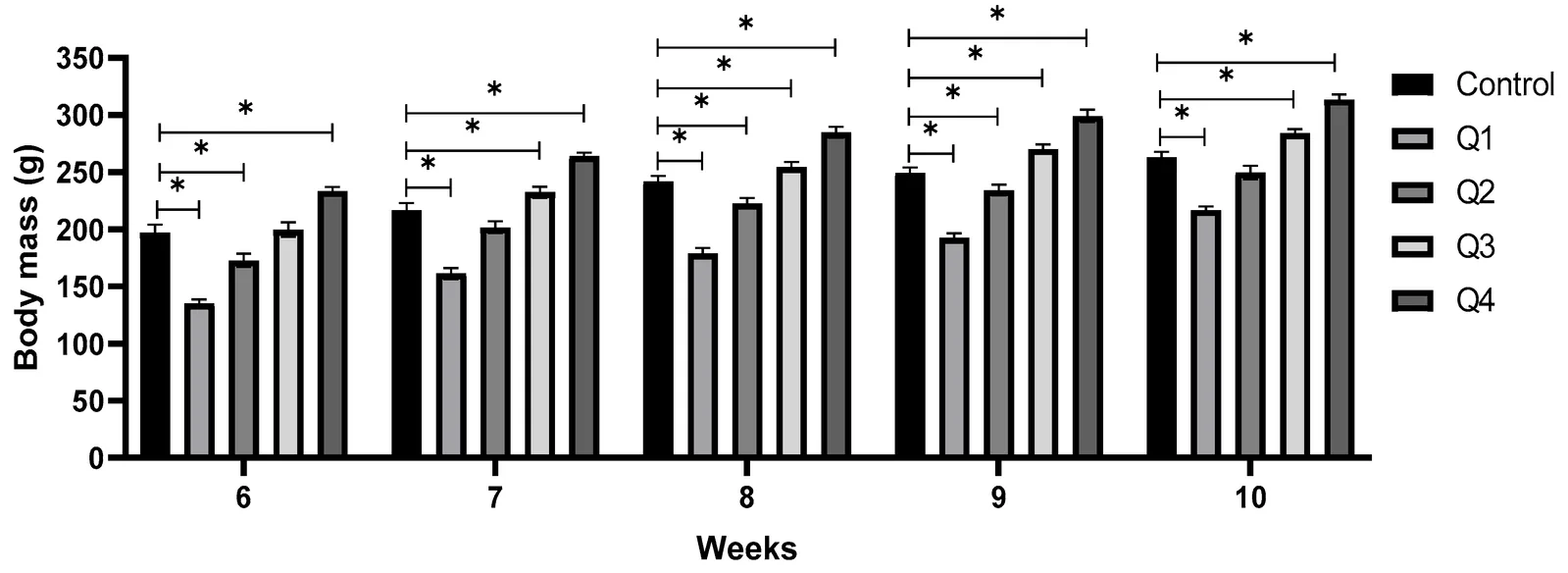
Effect of the cafeteria diet on rats’ body mass (g). Animals from the control groups were fed standard chow while animals from quartiles received cafeteria diet for 10 weeks (control n = 10, Q1 n= 7, Q2 n = 8, Q3 n = 7 and Q4 n = 7). Only the last 5 weeks are presented based on our previous publication [12]. Two-way ANOVA with repeated measures of Fisher’s LSD * *p* ≤ 0.05.

Figure 2 shows the body mass across the weeks observed in both control groups fed with standard pellets and the cafeteria-diet group (DCAF). Based on our previously reported results [12], in which body mass weight was significantly different between quartiles from the 6th week onwards, as shown in Figure 2, only the last 5 weeks (out of a 10-week-long experiment) are presented. We can see a larger increase in body mass in Q3 and Q4 compared to the control in the last 4 weeks. Interestingly, Q1 body mass remained consistently lower than that of the control group despite ad libitum access to the hypercaloric cafeteria diet. Q2 body mass was also lower than control during some weeks. Altogether, these data indicate that a subset of rats (Q1) fails to gain weight to the same extent as control animals or the other quartiles (Q2–Q4), regardless of the underlying cause. Based on previous results [12], Q1 showed a delayed weight gain during the weeks of the cafeteria diet compared to other quartiles.

### 3.2. Open Field Test

**Figure 3:**
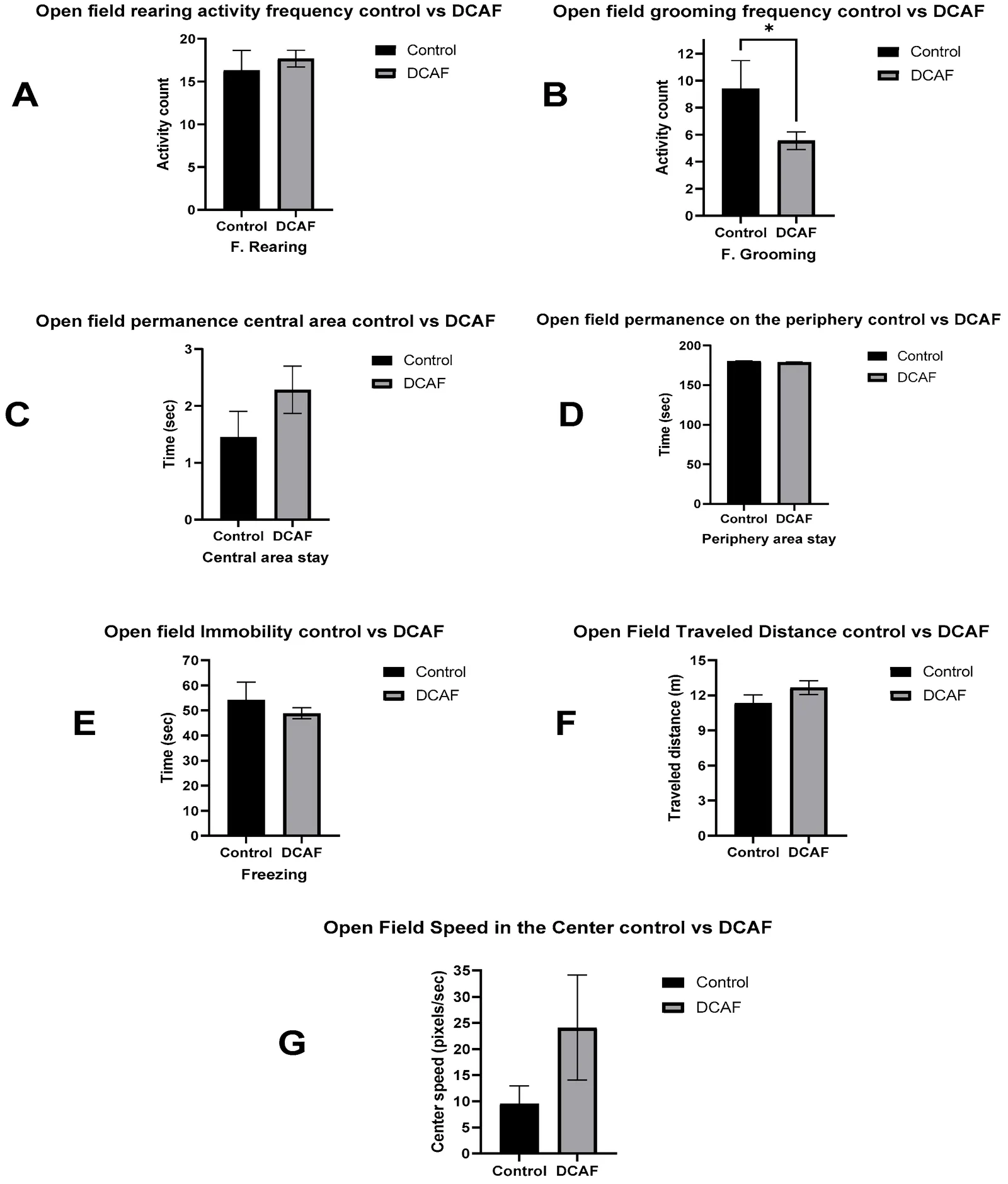
Effect of the cafeteria diet (DCAF) on anxiety-related behavior in the open field test, (**A**) shows rearing; (**B**) shows grooming, which is the only significant parameter; (**C**) shows the time spent in the central area; (**D**) shows the time spent in the peripheral area; (**E**) shows the freezing time; (**F**) shows the distance traveled; and (**G**) shows the speed in the center of the paradigm. Control and DCAF groups were compared using Student’s *t*-test or the Wilcoxon test, according to data distribution assessed by the Kolmogorov–Smirnov test. Data are presented as mean ± SEM. *p* < 0.05 was considered statistically significant (* *p* < 0.0321). Control, n = 10; cafeteria diet (DCAF), n = 29.

Next, we evaluated anxiety-like behaviors in rats exposed to a cafeteria diet vs. a control one. Figure 3 displays the effects of the cafeteria diet on rearing (Figure 3A), grooming (Figure 3B), time spent in the central area (Figure 3C), time spent in the peripheral area (Figure 3D), immobility (Figure 3E), distance traveled (Figure 3F), and speed in the center (Figure 3G) in the open field paradigm. There was a significant difference observed only in grooming behavior (Figure 3B), which was lower in cafeteria-diet animals compared to control animals (*p* ≤ 0.05). No significant differences were observed in all other parameters tested (*p* > 0.05 in all cases).

### 3.3. Hypothalamic Proteomics

A total of 1294 proteins were identified, and one-way ANOVA showed that 302 differed across groups (*p* ≤ 0.05). After FDR correction, 18 proteins remained significant (17 after excluding keratin 1; Table 1). Table 1 and Table 2 present the 17 FDR-supported proteins (one-way ANOVA with Benjamini–Hochberg correction, FDR ≤ 0.05; keratin 1 excluded), indicating which quartile differed from control (Tukey-adjusted *p* ≤ 0.05). The possible functions described in Table 2 are largely inferred from studies in other tissues (e.g., adipose tissue, pancreas, skeletal muscle, heart) and should be regarded as hypotheses rather than demonstrated functions in the hypothalamus. Pairwise post hoc analysis revealed that 181 proteins differed in relative abundance between Q1 and control rats, 52 between Q2 and control, 79 between Q3 and control, and eight between Q4 and control (Tukey-adjusted *p* ≤ 0.05; Table A1, Table A2, Table A3 and Table A4). As detailed below, global analysis of all quantified proteins showed greater proteomic similarity between Q4 and control and a greater difference between Q1 and control, a pattern also illustrated by heat maps restricted to ANOVA-significant and FDR-supported proteins.

**Figure 4:**
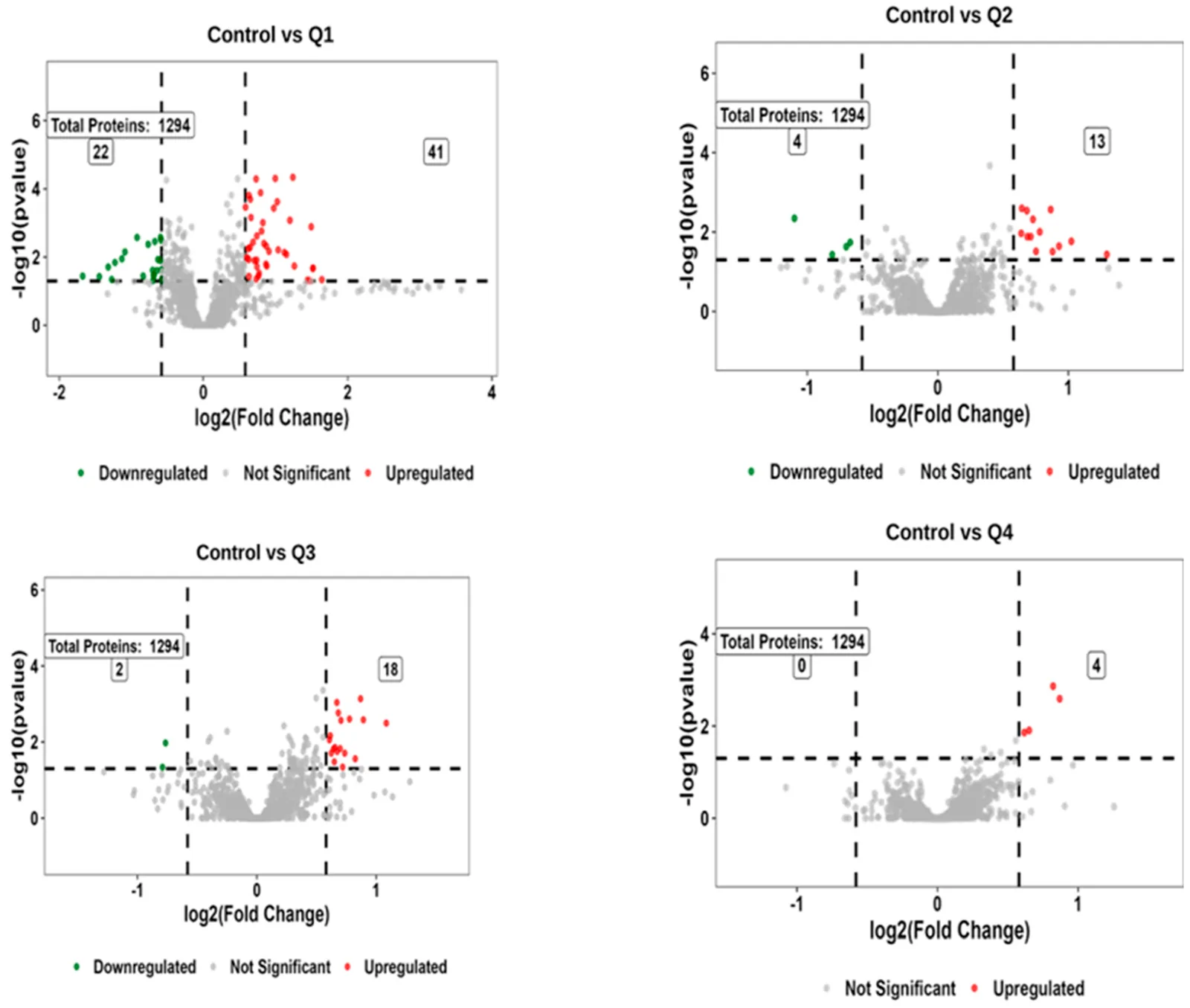
Volcano plots of hypothalamic protein abundance in each weight-gain quartile versus control (1294 proteins): Q1 (lower-weight-gain phenotype, n = 7), Q2 (n = 8), Q3 (n = 7) and Q4 (higher-weight-gain phenotype, n = 7); control n = 10. The horizontal dashed line indicates *p* = 0.05, and the vertical dashed lines indicate the fold-change threshold. Red, higher abundance; green, lower abundance, relative to control.

Since one of the objectives of our study was to determine whether there is a hypothalamic protein abundance profile associated with the lower-weight-gain phenotype, we first focused on the data set comparing the lower-weight-gain phenotype (Q1) to control animals (standard chow). Although control rats were not exposed to the obesogenic challenge, making it impossible to directly determine their underlying susceptibility to diet-induced weight gain, it is plausible that the control group also contained individuals with divergent susceptibility to weight gain, with this trait remaining masked under the non-obesogenic standard diet. This comparison evidenced 181 proteins that were significantly different (adjusted Tukey *p* ≤ 0.05; Figure 4, Table A1). It is likely that both diet itself and/or weight gain could have modified relative protein abundances. In this comparison, the 181 proteins could have encompassed both a candidate profile associated with the lower-weight-gain phenotype and protein abundance changes caused by diet.

**Figure 5:**
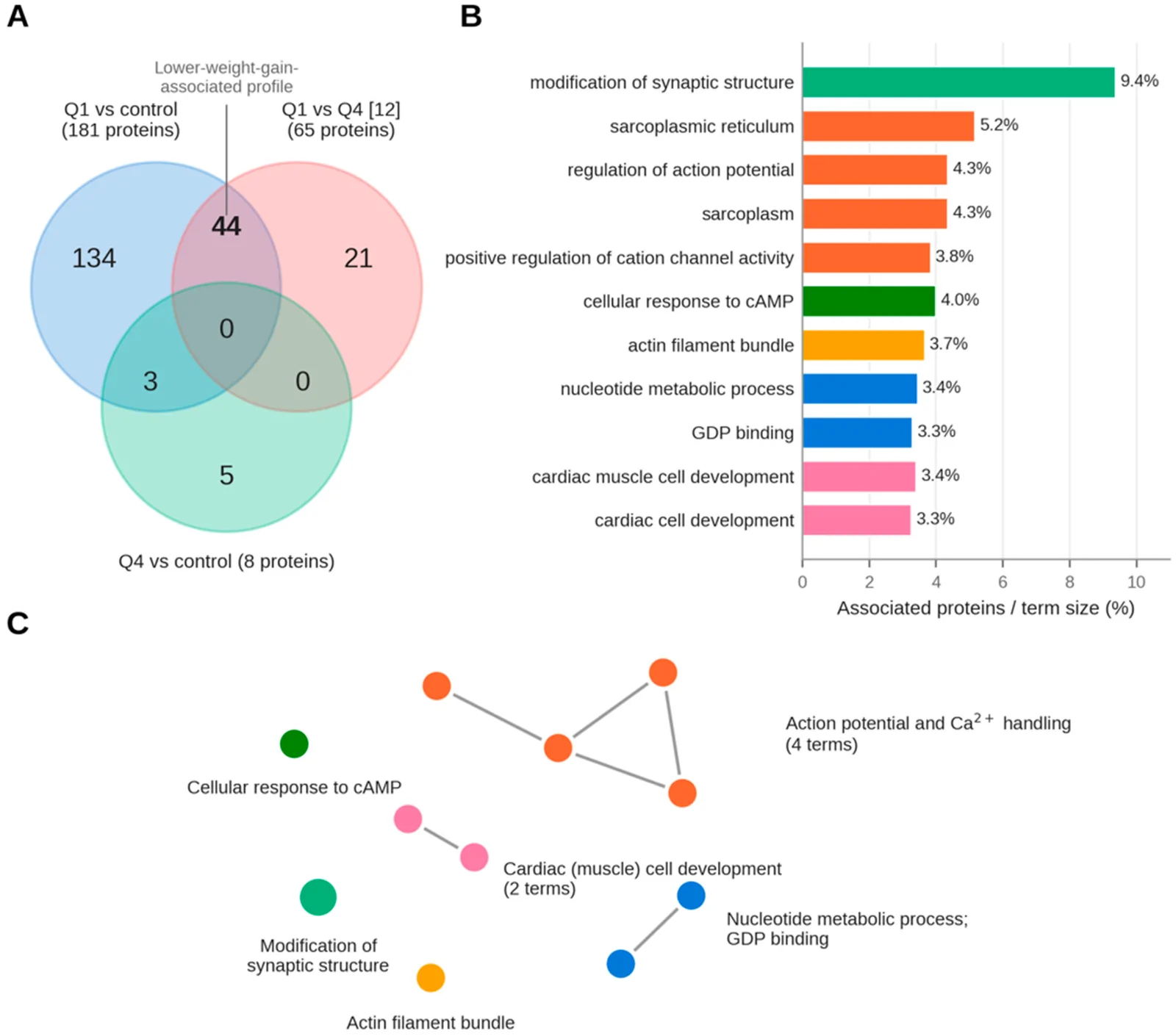
Set analysis and functional enrichment of the lower-weight-gain-associated profile. (**A**) Venn diagram showing the overlap among proteins significantly different (Tukey-adjusted *p* ≤ 0.05) in the Q1 vs. control comparison (181 proteins; this study), the Q1 vs. Q4 comparison (65 proteins; previous study [12]), and the Q4 vs. control comparison (8 proteins; this study). The 44 proteins shared by the Q1 vs. control and Q1 vs. Q4 comparisons constitute the lower-weight-gain-associated profile, which was used for functional enrichment analysis. (**B**) Enriched functional terms (*p* ≤ 0.05) for the 44-protein set, expressed as the percentage of each term’s annotated proteins present in the set. (**C**) The same terms are displayed as a ClueGO network, in which nodes represent terms, edges connect terms that share proteins (kappa score ≥ 0.5), and colors indicate functional groups, matching the bar colors in (**B**). Enrichment was performed in Cytoscape (v3.10.4) with the ClueGO app (v2.5.10). Q1, lower-weight-gain phenotype (n = 7); Q4, higher-weight-gain phenotype (n = 7); control, n = 10.

In a previous study [12], we compared exclusively Q1 vs. Q4, the lower- and higher-weight-gain phenotypes, respectively. In that data set, 65 proteins were significantly different between Q1 and Q4, and could have represented both the candidate lower-weight-gain-associated profile and protein abundance changes attributable to weight gain. In that comparison, diet-related changes could not be identified, since both Q1 and Q4 were maintained on the same diet, and the only difference between quartiles was body weight. Following this logic, by contrasting the 181 significantly different proteins between Q1 and control (this study) with the 65 significantly different proteins between Q1 and Q4 (previous publication; [12]), we reasoned that the overlap between these two data sets would be enriched for protein changes potentially involved in mechanisms of lower weight gain, while excluding changes attributable primarily to diet or to weight gain alone (Figure 5; Table A5). 

**Figure 6:**
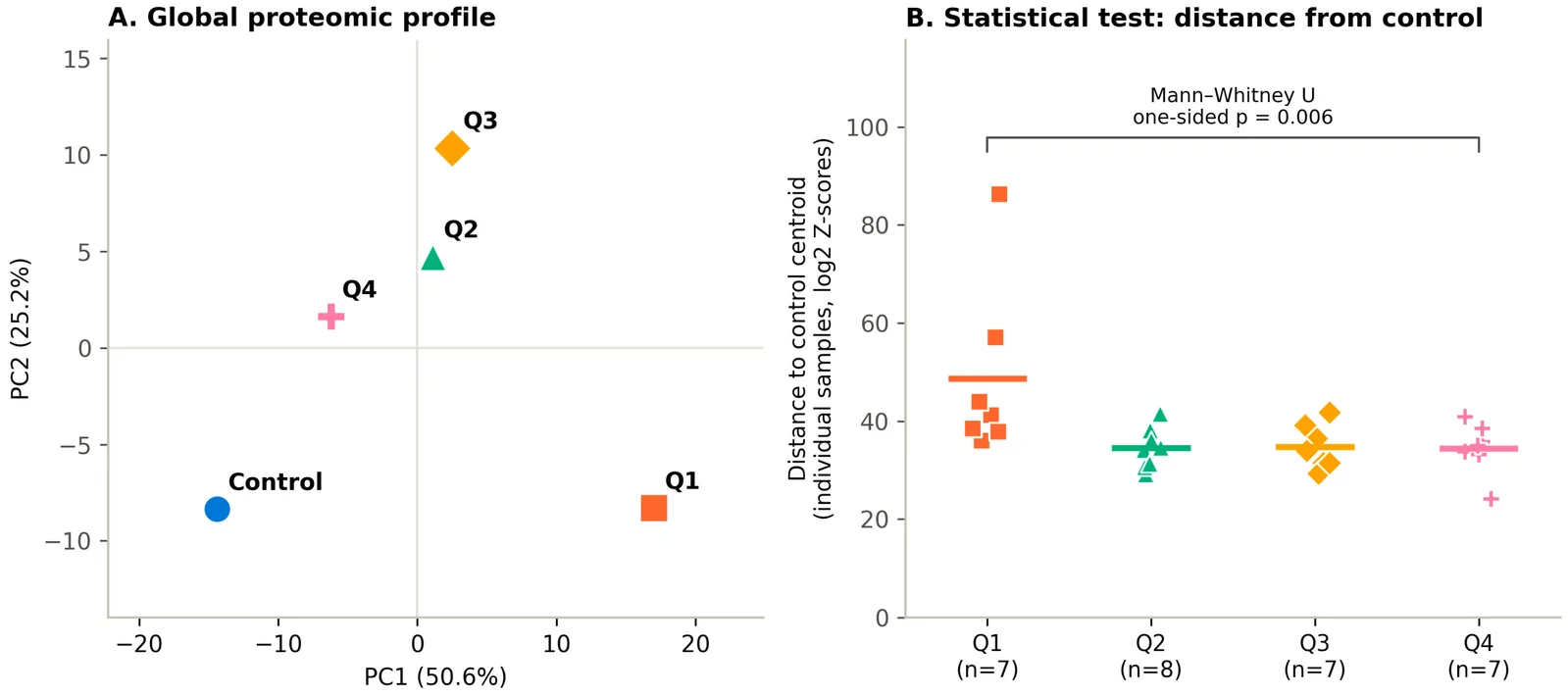
Global proteomic structure and quantitative divergence from control. (**A**) Principal component analysis (PCA) of mean relative protein abundance profiles (Z-scores) for control and each weight-gain quartile (Q1–Q4), summarizing the same underlying dataset as the hierarchical clustering in Figure A1. (**B**) Euclidean distance to the control group centroid (computed from 9 control samples) for each of the 29 individual biological replicates in quartiles Q1–Q4 (log2-transformed, per-protein Z-scored relative abundance; 1282/1294 proteins with complete data). One control sample was excluded during quality control because its number of missing values exceeded that of the established threshold for reliable assessment.

We then examined the Q4-vs-control comparison (Table 1) as a complementary test. Like the Q1-vs-control comparison, Q4 also differed significantly in body weight from control but in opposite direction; consequently, this comparison reflects the combined effects of diet and weight gain, and if there is a proteome related to lower-weight-gain (potentially present in control), this will unlikely be highlighted it with the same specificity. With this caution, the overlap between Q4-vs-control and the candidate lower-weight-gain profile was minimal, which is consistent with, but not independent confirmation of, our interpretation. The small size of this dataset (eight candidate proteins between control and Q4 by Tukey, of which only one, S1pr1, was FDR-supported; Table 1) could also suggest that the higher-weight-gain phenotype (Q4) shares more similarity with the overall population represented by control than does the lower-weight-gain phenotype (Q1), an idea further supported by the global analyses presented below (e.g., Figure 6). 

**Figure 7:**
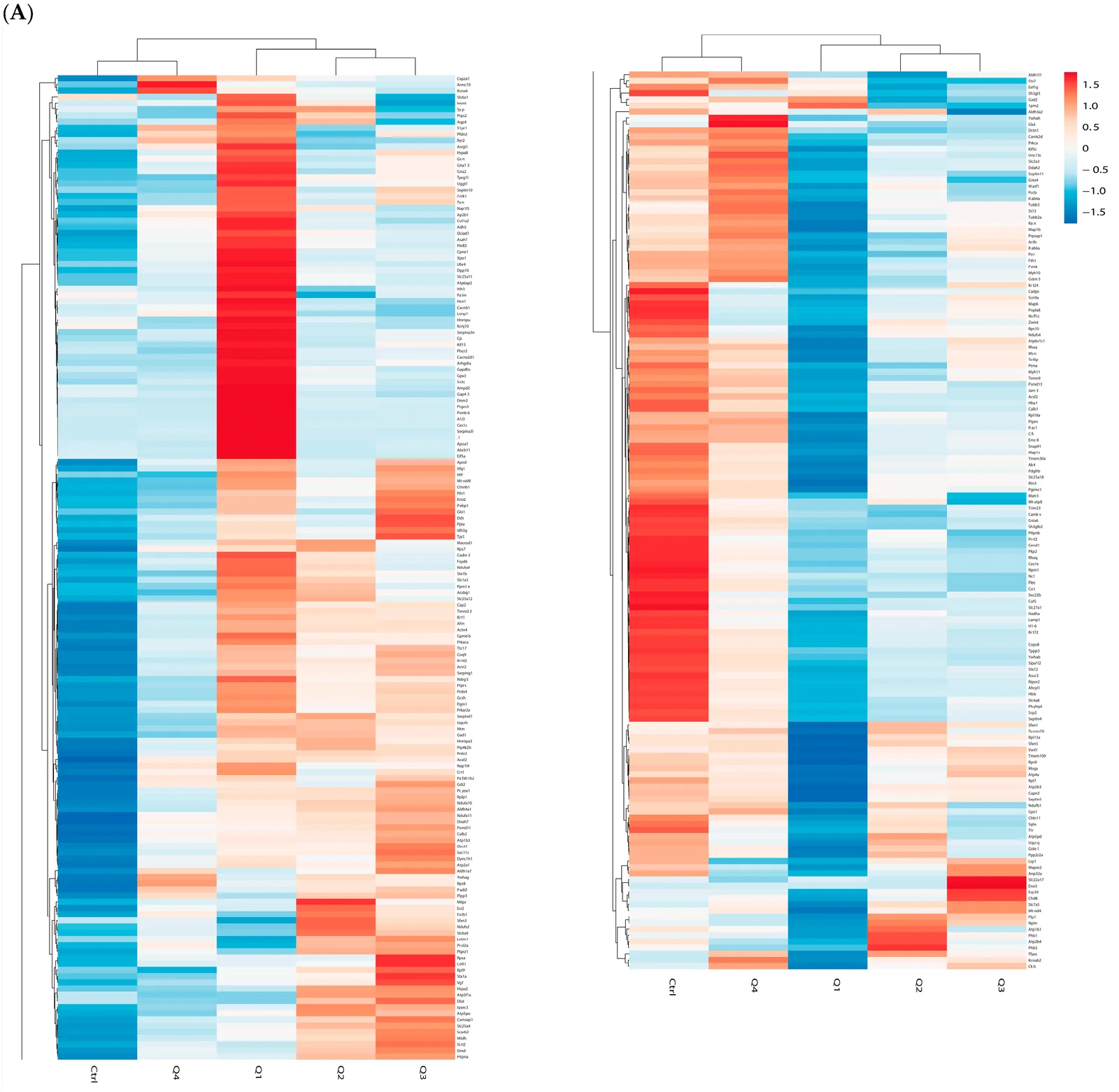

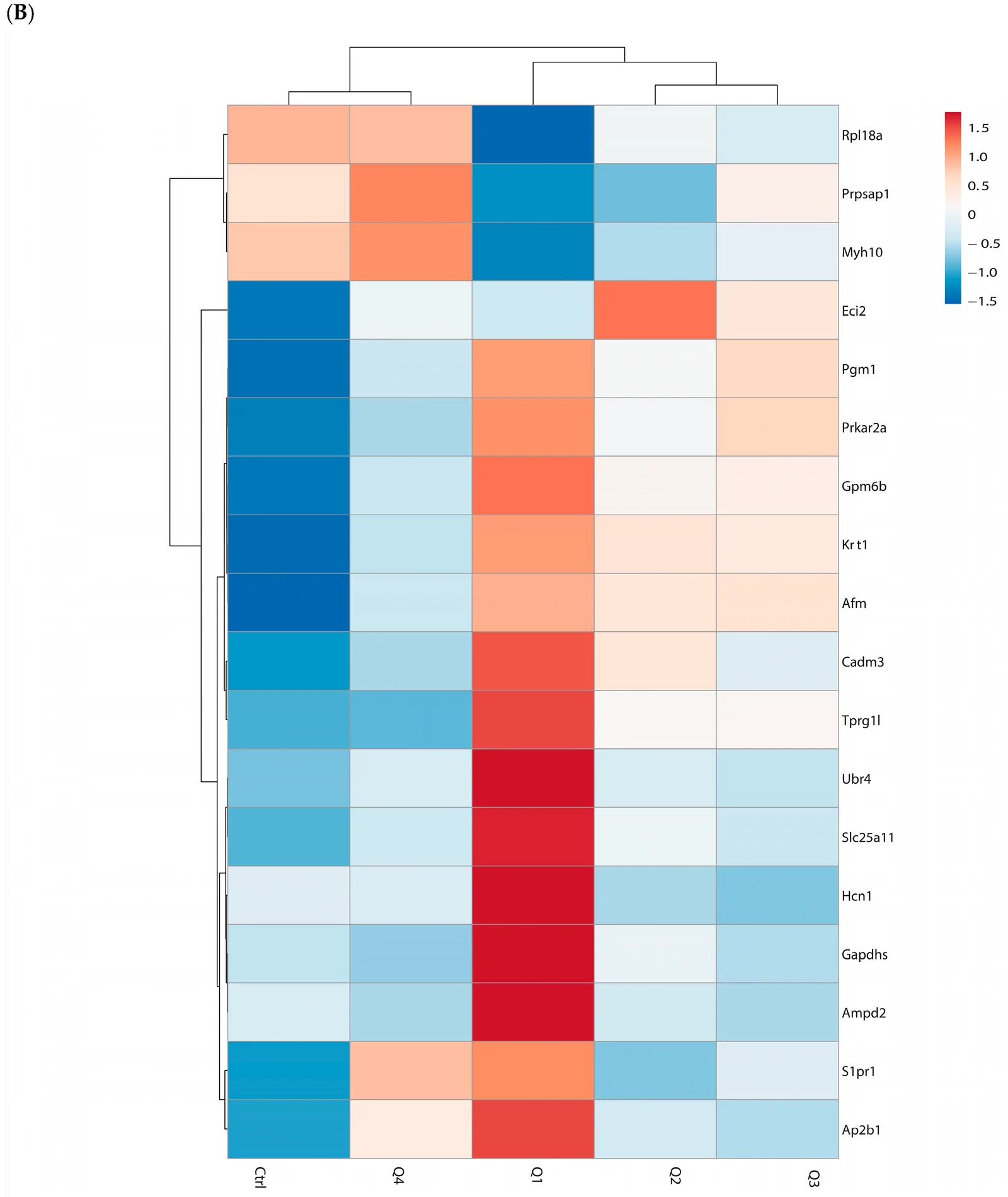
Hierarchical cluster heat maps of (**A**) 302 proteins with ANOVA *p* ≤ 0.05 and (**B**) the 17 FDR-supported proteins (Table 1), for control and each weight-gain quartile. Because these proteins were selected for differing among groups, these heat maps illustrate, rather than independently test, the pattern observed in the global analyses (Figure 6 and Figure A1).

**Figure A1:**
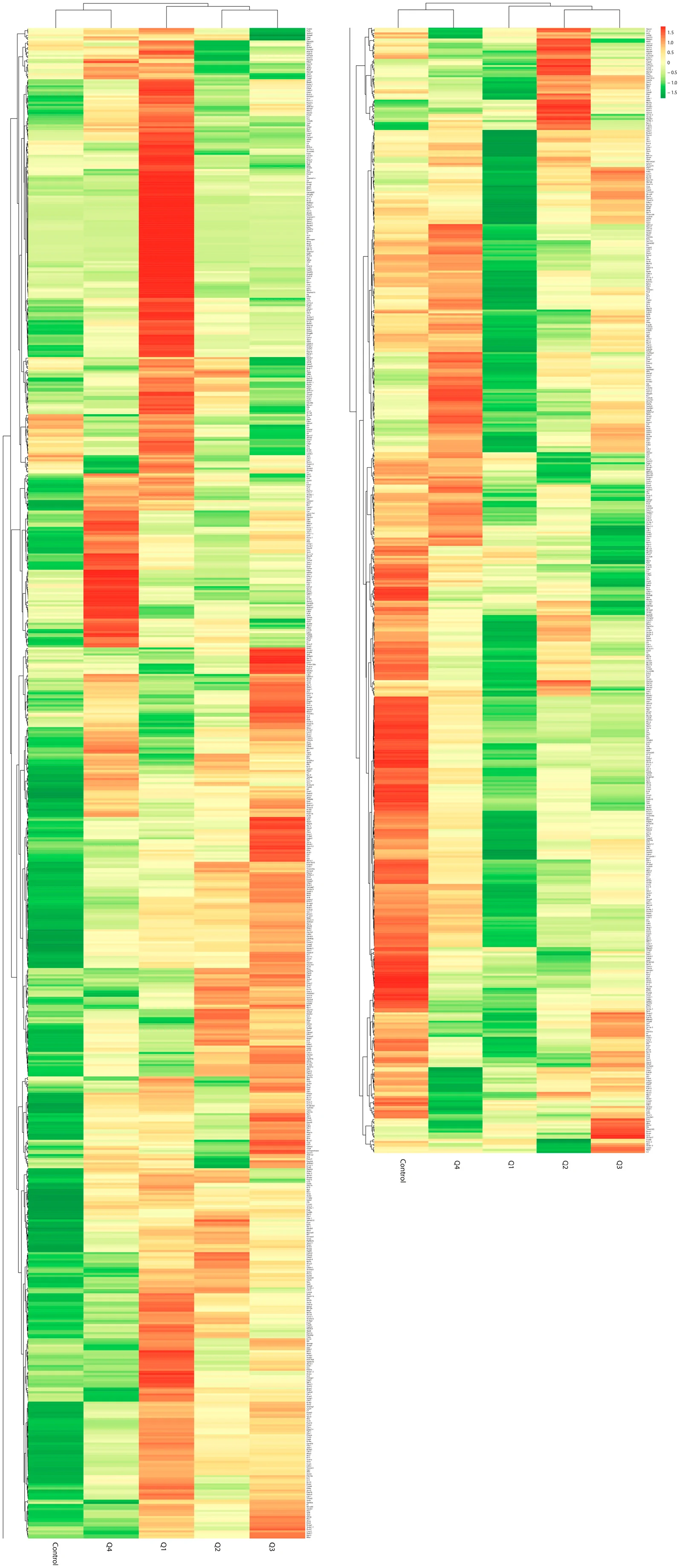
Hierarchical cluster heat map. Relative abundance data (1294 proteins) were transformed to raw Z-scores (protein variation across groups) and used for hierarchical clustering analysis, with correlation distance and average linkage as the clustering algorithm. The heatmap was generated in the “ClustVis” web-based tool [24].

When we examined relative protein abundance data globally, rather than only the significantly different proteins from pairwise comparisons (e.g., Q1 vs. control, Q4 vs. control), we observed two primary clusters: one comprising Q1, Q2, and Q3, and the other comprising control and Q4 (Figure A1). Within the first cluster, Q1 formed the most distinct branch, remaining closer to Q2–Q3 than to the control–Q4 cluster. Therefore, regardless of diet and weight gain, there were differences that set groups apart, with Q4 being closer to control than to the other quartiles, and Q1 being the most dissimilar to control. To further examine this global pattern, we performed a principal component analysis of the group-mean profiles (Figure 6A) and, to test it formally, calculated each sample’s multivariate distance to the control centroid using the complete individual-replicate dataset (Figure 6B). Consistent with the group-level clustering, PCA showed a clear separation of Q1 from control (Figure 6A), data that confirms and reinforces the findings reported in the present study. Q1 samples were significantly more distant from the control centroid than Q4 samples (mean distance 48.7 vs. 34.5 in standardized protein space; Mann–Whitney U, one-sided *p* = 0.006; Figure 6B), providing direct statistical support for the greater global proteomic divergence of the lower-weight-gain phenotype. Heat maps restricted to the 302 ANOVA-significant proteins and the 17 FDR-supported proteins showed the same clustering pattern (Figure 7).

In a parallel analysis, we compared the proteins most significantly upregulated and downregulated in the different quartiles compared to control (Table 1). Interestingly, several of these proteins were part of the candidate protein abundance changes, related to the lower-weight-gain profile observed in Figure 5.

The 44 proteins from Figure 5, which could indicate pathways or protein changes associated with lower weight gain, were analyzed by functional enrichment analysis. Functional enrichment analysis was performed in both the DAVID bioinformatics tool [25,26] and Cytoscape version 3.10.4 and its ClueGo app version 2.5.10 [22,23]. The functional annotation clustering obtained in the DAVID bioinformatics tool pointed out terms clustered as follows: “neutrophil degranulation”, “synapse”, “mitochondrion inner membrane”, “monoatomic ion transport”, “pathways of neurodegeneration”, and “nucleotide binding”. The graphical representation of the enriched functional terms is presented in Figure 5B,C, as a chart and network map, generated by Cytoscape and the ClueGo app. Both bioinformatic tools highlighted closely related functional terms. For instance: “modification of synaptic structure (Cytoscape) and “synaptic vesicle membrane” (DAVID), or “regulation of action potential” (Cytoscape) and “monoatomic ion transport” DAVID. Thus, both analytical tools were informative and complementary. Regarding the analysis shown in Figure 5B,C, several proteins were enriched in clustered terms (same color nodes), such as *Cacna2d1*, *Tomm70*, *Tmem109*, *Prps2*, and *Ran*, or across different clusters (different colors), such as *Camk2d*, *Hcn1,* and *Myh10*.

**Figure A2:**
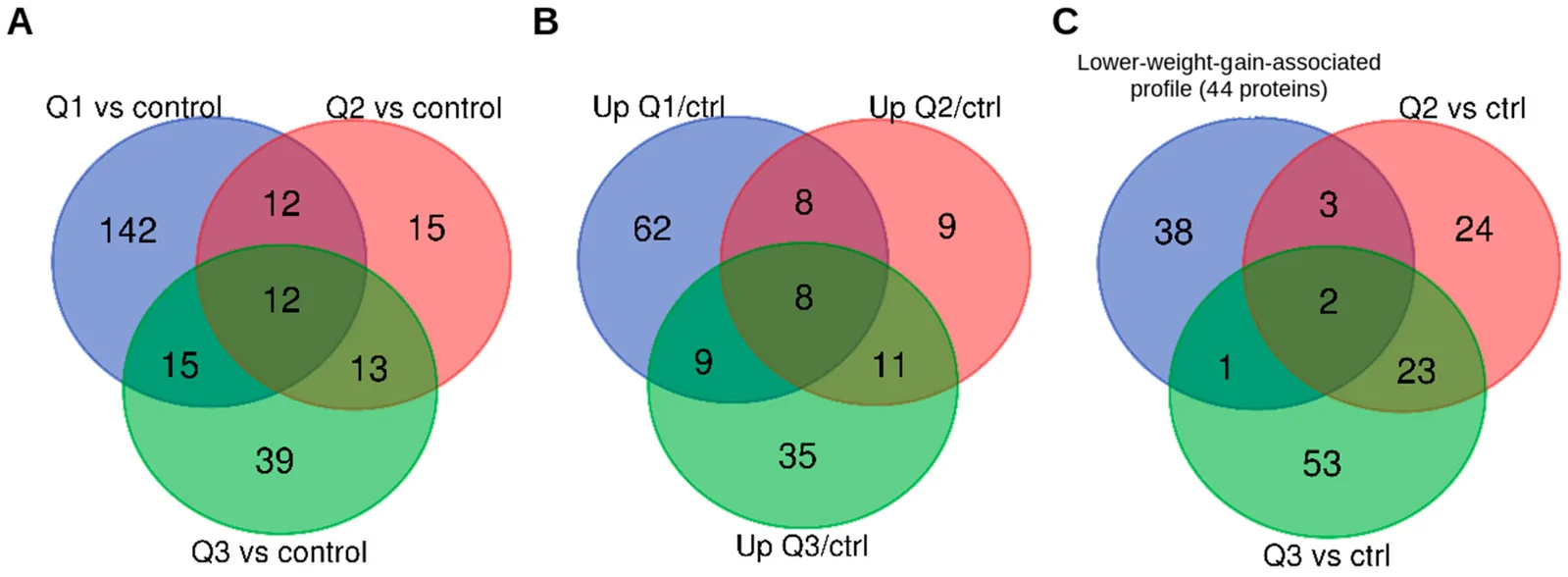
Set analysis of significantly different expressed proteins between groups. Venn diagrams showing the comparison of significantly different proteins between Q1, Q2, and Q3 vs. control (**A**); upregulated proteins in Q1, Q2, and Q3 vs. control (**B**); and the overlap between the lower-weight-gain-associated subset (44 proteins) and the significantly different proteins in the Q2 vs. control and Q3 vs. control comparisons (**C**).

To support this analysis, we also considered the Q2 vs. control and Q3 vs. control comparisons (Figure A2). Throughout the experiment, Q2 gained weight similarly to control rats fed standard chow, whereas Q3 showed a moderate increase relative to control, consistent with every ascending quartile representing progressively higher weight gain from Q1 to Q4. Accordingly, the proteins significantly different between Q2 and control (adjusted Tukey *p* ≤ 0.05) largely overlapped with those identified in the Q1 vs. control comparison, whereas the Q3 vs. control comparison yielded a more dissimilar set of significantly different proteins relative to Q1 vs. control (Figure A2). Therefore, although the proteomic differences in Q3 vs. control were large (79 significantly different proteins, Tukey-adjusted *p* ≤ 0.05), we hypothesized that those differences are categorically and functionally different in that comparison than in the Q1 vs. control (Table 1 and Table 2). The data from such comparisons are presented as Venn diagrams in Figure A2. Following this logic, we observed a higher percentage overlap in significant protein differences of Q1 vs. control (181 proteins) compared to Q2 vs. control (52 proteins) than when compared to Q3 vs. control (79 proteins). In other words, 46% of Q2 vs. control differently expressed proteins matched Q1 vs. control, and only 34% of Q3 vs. control proteins matched the Q1 vs. control proteome. Looking only at upregulated protein differences (Figure A2B), the conclusion is the same: there is a higher degree of similitude between Q1 and Q2 than between Q1 and Q3, as expected. This analysis matches the conclusion of the hierarchical clustering heat maps, considering the relative abundance of the 1294 proteins identified (Figure A1), the 302 significantly different proteins (ANOVA *p* ≤ 0.05), the 17 ANOVA FDR-corrected proteins (Figure 7), and the PCA in Figure 6A.

Interestingly, the suggested lower-weight-gain-associated profile (44 proteins) was compared to the data sets of Q2 vs. control and Q3 vs. control (Figure A2C). Almost no overlap was observed when comparing these data; for instance, only five of 52 proteins (9.6%, Q2 vs. control) matched the candidate lower-weight-gain-associated profile (44 proteins), while three of 79 proteins (3.8%, Q3 vs. control) matched this data set. This difference can be clearly observed in the heat maps of Figure 7. Notably, several proteins whose function has been partially characterized in heart physiology are highlighted in the lower-weight-gain-associated profile dataset, especially proteins involved in intracellular Ca^++^, Na^+,^ and K^+^ regulation.

## 4. Discussion

The results of the present study confirm that exposure to a cafeteria-type diet generates a marked phenotypic heterogeneity in body weight gain, even under controlled environmental conditions. This finding is consistent with the literature describing diet-induced obese (DIO) and diet-resistant (DR) phenotypes in rodent models, suggesting an intrinsic biological basis that modulates susceptibility to weight gain [11,46,47]. In this study, we included 10 control animals, studied simultaneously with the cafeteria-fed rats and maintained on standard chow, and compared their weight gain, behavior, and hypothalamic proteome to the divergent weight-gain phenotypes Q1–Q4 defined post hoc, induced by the cafeteria diet. The objective was to identify functional and molecular traits that could be associated with both higher and lower weight gain. By dividing the animals by weight quartiles, we aimed to enrich groups with both characteristics. We observed that Q1 animals maintained a weight equal to or less than the control despite the cafeteria diet, while Q4 animals significantly increased their weight (Figure 2). Cafeteria-fed animals showed reduced grooming compared with controls, whereas no significant differences were detected in locomotor, exploratory, or anxiety parameters measured in the open field test. This suggests that anxiety-like behavior was not markedly altered by the diet [16]; however, the open field test does not directly assess reward-related processes, and other behavioral tests could have provided additional information. In contrast, proteomic analysis of the hypothalamus revealed a significant difference between the groups, where the lower-weight-gain phenotype (Q1) showed 181 significantly different expressed proteins (Table A1) compared to the control animals, while the higher-weight-gain phenotype (Q4) showed only eight, of which only one (S1pr1) was FDR-supported (Table 1). Hierarchical cluster analysis of protein relative abundances in all quartiles and the control group pointed out that Q1 was more dissimilar to control rats than Q4 rats, despite both groups being fed a cafeteria diet and showing significant body mass differences to the control in opposite directions (Figure 6). Interestingly, the heat map of Figure A1 indicated that Q1, Q2, and Q3 were all further apart from the control than Q4. This finding suggests that rats of a higher-weight-gain phenotype maintained under a cafeteria diet show a relatively low difference from the overall population (control); and that the lower-weight-gain phenotype could involve extensive proteomic shifts from the average, consistent with the idea that weight gain is a common metabolic regulatory response to a cafeteria diet, while a lower-weight-gain phenotype is less frequent [11,48].

Consistent with this idea, data from the “National Health and Nutrition Examination Survey, NHANES” from the US, has pointed out an increase in energy intake and obesity prevalence among adults in a 30-year period [49]. Interestingly, this study highlighted that increased energy consumption was higher in normal-weight as well as overweight and obese men, indicating the existence of mechanisms to resist weight gain in some individuals. It has also been observed that there exists a small proportion of individuals with very low BMI (<18) without feeding disorders and normal energy intake in the population [50]. Overfeeding studies in humans and animals have demonstrated the variable weight-gain response to food intake. Interestingly, it is very likely that genetics plays a major role in these divergent phenotypes since classical studies in monozygotic tweens exposed to overfeeding lead to higher variability between pairs than within them [51,52]. In this regard, the *ALK* gene was identified in a cohort of healthy thin human subjects, and it was later demonstrated to control weight-gain susceptibility through its expression in hypothalamic neurons and its role in the regulation of lipolysis [53]. Notably, in our functional enrichment analysis, we have also identified several proteins and pathways whose mechanistic role in weight regulation warrants further investigation. We must stress, however, that in our study we were not able to monitor individual caloric consumption, and the quartiles represent enriched phenotypes, rather than categories with objective borders, which is a limitation in our work. A further limitation is that we analyzed the hypothalamus as a whole rather than isolating individual nuclei; because this region comprises multiple nuclei with distinct, and at times opposing, roles in energy balance (e.g., the arcuate, paraventricular, and ventromedial nuclei), as well as multiple cell types (neurons, astrocytes, microglia, and endothelial cells), whole-hypothalamus proteomics may dilute or mask nucleus- or cell-type-specific protein changes. Bulk proteomics cannot determine whether an observed abundance change reflects altered cell composition rather than altered protein levels within a given cell type, nor can it establish that a change in abundance corresponds to a specific altered functional activity; this is particularly relevant to enrichment terms such as “neutrophil degranulation,” which may reflect immune-cell presence in the tissue rather than a neuronal or glial process. Nonetheless, we must stress the high-content data obtained and the global analysis of protein changes as a significant advance in the understanding of weight regulation, since it is a complex phenotype controlled by the intertwining of several central and peripheral molecular mechanisms.

Additional limitations should be considered. The behavioral analysis was exploratory: seven open field outcomes were tested separately, so the grooming finding may reflect a type I error and should be interpreted cautiously, and the association between behavioral outcomes and weight-gain phenotype was not tested. Although quartile membership was not significantly associated with cage identity (permutation test, *p* = 0.089), cage-level body weight and food intake were not recorded, so a potential cage effect on weight gain or protein abundance could not be modeled statistically. The 44-protein lower-weight-gain-associated profile was derived from two comparisons that share the same Q1 samples and requires validation in an independent cohort, ideally with pre-exposure measurements. Because individual food intake, body composition, energy expenditure, and endocrine or metabolic status were not measured, the lower- and higher-weight-gain phenotypes are defined solely by body mass. Finally, only male rats were studied, so these findings cannot be generalized to females.

### 4.1. Effects of the Cafeteria Diet on Body Mass: Weight Gain by Quartiles

Body mass graphs (Figure 2) show that after several weeks of cafeteria dieting, rats in Q4 (upper quartile) showed a significant increase compared to controls with a standard diet (* *p* < 0.05), while Q1 (lower quartile) remained close to or below the control. As already mentioned, of the 30 rats exposed to the diet, a typical pattern is observed, where only a fraction of the rats become overweight. This pattern coincides with previous literature; for example, Xi and colleagues reported that in rats fed a high-fat diet (4 months), more than 50% of rats will develop diet-induced obesity (DIO), while approximately 20% remain resistant [11]. Thus, our empirical results reproduce the classic bifurcation between DIO and resistant subjects [47,48], i.e., given the same exposure to a hypercaloric environment, individuals differ in weight gain. Because all cafeteria-fed animals had ad libitum access to the same diet and were randomly allocated to cages, gross differences in dietary environment are unlikely to explain this divergence; however, individual food intake and cage-level effects were not quantified and cannot be excluded. Some animal studies suggest that weight gain may also depend on innate neuroendocrine responses, beyond differences in caloric intake [11,54]; because individual intake was not measured here, this possibility could not be tested. We did not include metabolic or endocrine profiles in our analysis, which could have helped to rule out possible mechanisms of weight-gain regulation.

### 4.2. Behavior and Anxiety in the Open Field Test

Open field analysis of behavior and anxiety revealed few differences between groups. No significant changes were detected in distance traveled or time in the central zone (*p* > 0.05), indicating the same level of exploration and locomotor activity between diets (Figure 3). The only finding was a reduction in grooming in the cafeteria-diet group compared to the control group, suggesting perhaps a higher stress or anxiety experienced by cafeteria-diet fed animals, which is in line with previous evidence of cafeteria-diet-induced central inflammation and anxiety [5] and binge eating behavior [55]. However, we should interpret our data carefully, as grooming has been shown to be indicative of different behaviors and change in a context-specific manner [56]. This is consistent with the review by [16]; according to these authors, to date, no clear effects of the cafeteria diet on locomotor activity, impulsivity, or social behaviors have been conclusively drawn.

Similarly, other experimental studies report that the behavioral effects of obesogenic diets may be subtle and paradigm-dependent [5]. In that work, a 2-week cafeteria diet induced increased anxiety in elevated cross maze tests, but in our study, the open field test was less sensitive (only grooming changed). Therefore, the findings suggest that high/low weight gain was not correlated with evident alterations in exploration or anxious behavior in the open field, which reinforces that other mechanisms (neurobiological/metabolic) are dominant.

### 4.3. Response of Hypothalamic Proteomics to Induce Weight Gain

The most notable finding was the marked asymmetry in hypothalamic protein abundance divergence from control between the two phenotypes. In the LC–MS/MS analysis, 1294 total proteins were identified; of these, 181 differed significantly between Q1 and control (Table A1), while only eight did so between Q4 and control, of which only one (S1pr1) was FDR-supported (Table 1). Our results complement previous studies, in which only 31 significantly different expressed proteins were observed in the hypothalamus of diet-induced overweight vs. overweight resistant rats [11]. Thus, our data suggest that the lower-weight-gain phenotype (Q1) could have larger functional and structural differences than the higher-weight-gain phenotype (Q4) when compared to the hypothalamus of control rats. The above may indicate that the lower-weight-gain phenotype involves an intrinsic molecular divergence that dominates the effect of diet and weight, while the higher-weight-gain phenotype would share more common molecular features in the hypothalamic proteome, with most of the population represented in control (hence only eight candidate changes, and one FDR-supported change, in Q4 vs. control; Figure 5), a hypothesis also supported by hierarchical cluster analysis (Figure A1), and PCA (Figure 6A), and other research groups [47,57]. This result is consistent with epidemiological data discussed earlier and the evolutionary hypothesis, in favor of the existence of a dual point selective pressure that safeguards normal body weight, in which the upper limit (excess body weight) has been lost since predator avoidance has become unnecessary and therefore it is easier to find overweight than underweight individuals [48]. Because all control animals were housed in a single cage, a cage effect cannot be formally separated from the control condition. Nevertheless, such an effect would be expected to influence all quartile versus control comparisons similarly, whereas only Q1 diverged markedly from control, while Q2–Q4 remained close to it (Figure 6). This pattern is therefore unlikely to be explained by housing alone, although the single control cage remains a limitation of the design.

Functional enrichment analysis indicates changes in neuronal plasticity and mitochondrial function in the lower-weight-gain phenotype rats, as evidenced by increased expression of *Hcn1*, *Stx1b*, *Ryr2*, and *Dpp10*, for example. This could indicate that hypothalamic neurons of Q1 rats have greater excitatory and calcium-modulated transmission than control hypothalamic neurons. Similarly, the elevation of *Ndufa9* and the relative decrease in *Atp5pd*/*Tomm70* may reflect disruption in hypothalamic energy metabolism. At the same time, changes in *Camk2d* and *Vsnl1* suggest additional regulation in Ca^2+^ signaling and synaptic plasticity. In line with our results, studies in humans have revealed microstructural changes in the hypothalamus of obese individuals [58], while animal studies have pointed out that increased intracellular Ca^++^ levels disrupt the excitability of hypothalamic neurons, and this mechanism is induced by obesity [59], in agreement with the molecular process and protein changes observed in our work.

Along the same line, Xi and collaborators [11] showed that in obesity-resistant rats there was an enrichment of several proteins related to neuronal functions and mitochondrial biogenesis compared to obese rats, which the authors interpreted as more efficient regulation of energy balance in diet-resistant animals. Globally, we have allocated the significantly different expressed proteins between control and Q1 or Q4 in the following functional categories.

### 4.4. Proteins Related to Metabolism in the Hypothalamus

Unless otherwise indicated, the proteins discussed below are exploratory candidates from the Tukey-based comparisons (Table A1, Table A2, Table A3 and Table A4). Significantly different proteins with metabolic roles were observed in Q1 vs. control, but not in Q4 vs. control. The upregulated proteins in Q1 compared to control were *Slc25a12*, *Ndufa9*, and *Asrgl1*; whilst the downregulated ones were *Tomm70*, *Atp5pd*, and *Ndufb1*.

The hypothalamic solute carrier family 25 (aspartate/glutamate carrier) member 12 (*Slc25a12*) increased in our study. This antiporter carrier imports glutamate and exports aspartate as part of the malate–aspartate cycle that generates reduced species such as NADH within the mitochondria [60], thus facilitating aerobic energy production in high-demanding tissues such as nervous structures, in which it is also involved in neurotransmitter homeostasis [61]. This may resemble increased nervous activity related to energy expenditure and metabolism. Interestingly, in structures out of the nervous system, such as skeletal muscle under pathological conditions (i.e., obesity and diabetes mellitus) and hypercaloric diet, a downregulation of this carrier is observed as a possible compensatory mechanism to prevent apoptosis due to the excess of lipids [62,63].

Other mitochondrial-related proteins significantly different between Q1 and the control were the NADH dehydrogenase (ubiquinone) 1 alpha subcomplex subunit 9 (*Ndufa9*) and the NADH dehydrogenase (ubiquinone) 1 beta subcomplex subunit 1 (*Ndufb1*). In a study of GLP-1 receptor loss of function in astrocytes, changes in complex I subunits were observed, with *Ndufa9* remaining intact, while another subunit, *Ndufb8*, was downregulated, and this latter has been related to complex I dysfunction [64]. In the brain, upregulation of *Ndufa9* is an adaptive response to the excess of energy substrates and metabolic stress in models of diet-induced obesity [65]. In the peripheral adipose tissue, *Ndufa9* overexpression participates in the conversion of white adipose tissue to brown adipose tissue involved in energy expenditure to heat production [66]. The downregulated *Ndufb1*, the ATP synthase subunit d (*Atp5pd*), and the mitochondrial import receptor subunit TOM70 seen in our study contrast with the upregulation of *Ndufa9,* and could be due to the inefficient transport of electrons in complex I (downregulated *Ndufb1*), a poor rate of ATP production by ATP synthase (downregulated Atp5pd), and a decrease in mitochondrial transporters involved in amino acid metabolism and thermogenic capability (i.e., ANT2 and SLC25A44), [67,68]. We cannot be sure whether the upregulation of *Ndufa9* of the mitochondrial complex I obeys a compensatory response to this hypothetical limited respiratory chain performance, as seen in other scenarios such as hypoxia, where respiratory chain is compromised, and *Ndufa9* is also upregulated [69].

The downregulated Isoaspartyl peptidase/L-asparaginase (*Asrgl1*) is a protease involved in the deamination of asparagine to aspartate [70]. Aspartate in the form of D and/or L isomers is scarce in the CNS; however, its presence seems to modulate feeding behavior by decreasing anorexigenic proopiomelanocortin and α-melanocyte-stimulating hormones [71]. It follows that the upregulation of Asrgl1 should increase feeding; however, its function in the hypothalamus is still incompletely characterized. In this regard, one of the limitations of our work was the absence of the quantification of food consumed, and thus further research needs to be done to elucidate in a more mechanistic way the interaction of the proteins exposed here and differentiate the effects attributed to metabolism from those related to feeding behavior.

### 4.5. Neuronal Excitability and Neuroplasticity

The number of significantly altered proteins in Q1 vs. control group was notably greater than Q4 vs. control. We observed the upregulation of Ryanodine receptor 2 (*Ryr2*), Syntaxin-1B (*Stx1b*), and inactive dipeptidyl peptidase 10 (*Dpp10*) and the downregulation of Visinin-like protein 1 (*Vsnl1*) in Q1 compared to control, while in Q4, we observed only the upregulation of voltage-gated potassium channel subunit Kv1.4 (*Kcna4*) compared to control.

Syntaxin-1B is involved in stabilizing the SNAP25-synaptobrevin in the t-SNARE multiprotein complex. The available information about Syntaxin-1B is scarce; however, in mice lacking the isoform STX1A, several hormones of the hypothalamic-pituitary axis reduced their levels [72]. Thus, an increase in the expression of these proteins or related to Syntaxin isoforms may facilitate the release of hypothalamic hormones, possibly including those related to metabolism and feeding behavior. In this sense, the observed upregulation of *Ryr2* (Ryanodine receptor 2) in Q1 is known for mediating intracellular calcium mobilization from the endoplasmic reticulum (ER) and may facilitate neurotransmission. In high-fat diet models, calcium homeostasis plays a regulative role via calcium calmodulin kinases (CaMKK) [73] and can induce orexigenic NPY activity [74]; however, under high-lipid diets, increased Ca^2+^ levels from ER have been shown to promote mitochondrial dysfunction in hypothalamic neurons [75]. Other proteins related to calcium dynamics in the Q1 individuals were the plasma membrane calcium-transporting ATPase 3 (*Atp2b3v*) and the Visinin-like protein 1 (*Vsnl1*). These proteins are functionally dedicated to the transport of Ca^2+^ from the cytosol to the extracellular matrix (i.e., *Atp2b3v*), [76,77], and sense intracellular calcium levels (i.e., *Vsnl1*). Interestingly, the presence of Vsnl1 has also been associated with brain damage biomarkers [78]; however, in our study, this protein was downregulated. This protein was among the 44 proteins that we hypothesize could be part of a regulatory network associated with lower weight gain, but whether its downregulation is functionally related to weight gain remains unknown.

Only the voltage-gated potassium channel subunit Kv1.4 was upregulated in the Q4 (higher-weight-gain) group (Table A4), and this may correspond to changes in excitability in the hypothalamic neurons as well as biomarkers for neuronal damage. Changes in the Kv channels have been associated with damage biomarkers in neurodegeneration and cell death. The case of Kv1.4 and others is increased in the brain of Aβ amyloid-infused animals [79]. It is well documented that obesity is a risk factor related to neurodegenerative diseases such as Alzheimer’s [80]. In this sense, the presence of upregulated Kv1.4 subunits in the higher-weight-gain Q4 group may represent an indicator of physiological stress in hypothalamic neurons.

### 4.6. Cytoskeletal and Cellular Trafficking

When comparing the lower-weight-gain phenotype with control animals, we identified several proteins involved in cytoskeleton dynamics and rearrangements, such as *Kif15*, *Ap2b1*, *Septin11*, *Myh10*, and *Wasf1*, most of which also belong to the 44-protein profile (Table A5). For example, Septin11 has been involved in neuritogenesis [81], while *Wasf1* participates in actin filament rearrangements. *Septin11* has been associated with lobar frontotemporal dementia, and in adipocytes has been shown to regulate lipid and glucose metabolism [82]. Therefore, it could represent the link between metabolism and structural changes associated with lower weight gain.

In addition, changes in *Kif15* and *Myh10* also highlight cytoskeletal reorganization differences in the lower-weight-gain phenotype animals compared to controls. In line with our results, Chen and collaborators [83] observed that the use of empagliflozin in obese mice produced changes in the phosphorylation of cytoplasmic proteins such as *Myh10*, and interestingly, these proteomic changes were associated with better spatial memory performance. In other words, by improving metabolic function in obese mice, phosphorylation of cytoskeletal proteins was reestablished together with cognitive performance [83]. We also observed changes in other proteins such as *Gpm6m*, a neuronal membrane glycoprotein that is hypothesized to participate in neuronal development. Indeed, a recent *Gpm6m* KO mouse was developed, and functional differences in the reward system were observed [31]. Also, as pointed out, we observed an increase in kinesin subunits (i.e., kinesin-like protein *Kif15*) relevant to intracellular transport, cell division, and remodeling of the cytoskeleton [84] and *Ap2b1*, a protein involved in the internalization of membrane receptors in a clathrin-dependent manner [85]. It has been reported that deficiency of *Kif15* leads to poor synaptic development [86], while the adaptor protein 2 (*Ap2*) plays a crucial role in axogenesis, stabilizing the polarity of the neuron [87], and regulating action potentials through synapse stabilization and controlling the population of glutamatergic receptors [88]. This means that the lower-weight-gain individuals upregulate proteins involved in membrane cytoarchitecture and synaptic functionality, which we hypothesize could contribute to lower weight gain under an obesogenic diet. Nonetheless, more studies are needed before a functional relevance of each of these proteins is clearly established in such model.

Together, these data suggest there could be differences in hypothalamic structure as part of the characteristics that may be associated with lower weight gain in rats. Notably, a recent study addressed the correlation of obesity, protein expression, and brain structure in humans [89], and found a positive association between cortical thinning and body mass index across different age groups [89]. Thaler et al. [9] have also found fast and sustained hypothalamic neuroinflammation upon a high-fat diet in rats and mice, as well as enhanced gliosis in obese people [9]. Other studies have also observed changes in the expression of microtubule-associated 1B (Map1B) in mice fed a high-fat diet [90], and other cytoskeletal proteins have been shown to be modified in the brain of obese animals [91]. Therefore, our data are in line with several proteomics studies that highlight changes in cytoskeletal proteins linked to obesity, as well as the functional relevance of these modifications, highlighting the impact and applicability of our analysis.

### 4.7. Integration

Hierarchical clustering, PCA, functional enrichment, and set analysis were employed to identify the molecular processes that are likely affected in Q1 and Q4 weight phenotypes compared to control rats. The above-highlighted proteins are documented to play important roles in the functional remodeling of neurons in the hypothalamus. There is a possibility that remodeling is multifactorial, comprising changes at metabolic, morphological, and functional excitable, neuroplastic, and nuclear levels. These changes are not commonly observed in the Q4 (higher-weight-gain) group, in agreement with the heat map of Figure A1 that highlights close similarity between Control and Q4, and further apart (even opposite changes) in relative protein abundances in Q1. The mechanism behind this differentiated response remains obscure and may have an evolutionary or epigenetic explanation.

## 5. Conclusions

In the cafeteria-diet model, the lower-weight-gain phenotype showed greater hypothalamic proteomic divergence from control animals than the higher-weight-gain phenotype, as revealed by global, threshold-independent analyses. Exploratory protein-level analyses suggest that this divergence may involve neuronal plasticity, mitochondrial bioenergetics, and excitability regulation, which remain hypotheses for future validation. Whether higher weight gain represents the predominant response to an obesogenic environment requires further study, and validation in female rats is necessary before these findings can be generalized across sexes. These results identify candidate neurobiological pathways that warrant further investigation in relation to differential weight gain.

## Figures and Tables

**Table 1:** FDR-supported proteins (n = 17) with significant differences across groups (one-way ANOVA with Benjamini–Hochberg correction, FDR ≤ 0.05; keratin 1 excluded as a probable contaminant). Pairwise differences versus control were assessed with Tukey’s post hoc test (adjusted *p* ≤ 0.05). The heading with a yellow background indicates the names of the genes for the proteins analyzed; the heading with an orange background indicates a significant difference between Q1 and the control; the heading with a blue background indicates a significant difference between Q2 or Q3 and the control; the heading with a green background shows a significant difference between Q4 and the control; gray-colored cells indicate no significant difference compared to the control.

Protein Name	Ratio Relative Abundance Q1/Control	Tukey-Adjusted *p* ≤ 0.05	Ratio Relative Abundance Q2/Control	Tukey-Adjusted *p* ≤ 0.05	Ratio Relative Abundance Q3/Control	Tukey-Adjusted *p* ≤ 0.05	Ratio Relative Abundance Q4/Control	Tukey-Adjusted *p* ≤ 0.05
*S1pr1*	1.966417	0.00037					1.827084	0.00256
*Ap2b1*	1.254529	0.00028						
*Pgm1*	1.995351	0.00005	1.608656	0.01307	1.825985	0.00073		
*Afm*	2.033235	0.00024	1.821144	0.00272	1.85631	0.00261		
*Gpm6b*	1.659035	0.00005	1.386016	0.01926	1.404675	0.01787		
*Prpsap1*	0.757839	0.00722	0.812574	0.04372				
*Prkar2a*	1.579838	0.00069			1.470123	0.00740		
*Cadm3*	1.569658	0.00020	1.349533	0.02724				
*Ubr4*	1.389685	0.00005						
*Slc25a11*	1.077743	0.00015						
*Myh10*	0.893777	0.00132						
*Ampd2*	1.548587	0.00016						
*Gapdhs*	2.362417	0.00005						
*Rpl18a*	0.700955	0.00006						
*Tprg1l*	1.499088	0.00035						
*Hcn1*	1.540951	0.00565						
*Eci2*			1.318059	0.00021	1.211059	0.02671		

**Table 2:** Potential functions of the 17 FDR-supported proteins listed in Table 1 (one-way ANOVA with Benjamini–Hochberg correction, FDR ≤ 0.05). The functions described are based on studies in diverse neural and non-neural tissues and are not necessarily specific to the hypothalamus.

Gene	Possible Function in Body Weight Regulation
*S1pr1*	Sphingosine-1-phosphate receptor 1. Regulates energy homeostasis and feeding behavior in the hypothalamus (S1P/S1P1/STAT3 pathway). Its alteration modulates white adipose tissue (WAT) expansion and inflammation and limits brown adipogenesis, impacting obesity [27].
*Ap2b1*	β subunit of the AP-2 adaptor complex, key in clathrin-mediated endocytosis; controls the internalization and recycling of hormone receptors such as insulin and leptin receptors. Its alteration could modify the hormonal signaling that regulates energy storage and appetite [28].
*Pgm1*	Phosphoglucomutase 1. Interconverts glucose-1-phosphate and glucose-6-phosphate, linking glycolysis and glycogenolysis. The PGM1*2 allele is associated with relative protection against extreme increases in body mass, owing to differences in carbohydrate utilization [29].
*Afm*	Afamin. Vitamin E-transporting glycoprotein linked to metabolic syndrome and insulin sensitivity. Serum levels correlate with BMI and waist circumference and drop sharply after severe weight loss (e.g., bariatric surgery), acting as a sensor of energy balance [30].
*Gpm6b*	Glycoprotein M6B. Expressed in the central nervous system; allosterically reduces serotonin transporter (SERT) activity at the cell surface. Because hypothalamic serotonergic pathways modulate satiety, energy balance, and macronutrient preference, Gpm6b alterations may indirectly modify susceptibility to weight gain [31].
*Prpsap1*	Phosphoribosyl pyrophosphate synthetase-associated protein 1. Negative regulator of phosphoribosyl pyrophosphate (PRPP) synthesis and nucleotide synthesis. Identified in a genome-wide analysis as a candidate gene associated with abdominal fat deposition in lean and fat chicken lines [32,33].
*Prkar2a*	PKA regulatory subunit RII-alpha. Its genetic deletion confers resistance to high-fat diet-induced obesity (DIO) by increasing thermogenesis in brown adipose tissue and modulating reward-related behavior toward high-calorie foods [34].
*Cadm3*	Cell adhesion molecule 3. Regulates synaptic assembly and axon–glia interactions. Belongs to the CADM family, whose members are upregulated in the hypothalamus in obesity and facilitate weight gain by reorganizing neuronal projections toward anorexigenic POMC and orexigenic AgRP cells [35].
*Ubr4*	E3 ubiquitin ligase component N-recognin 4. Involved in cellular protein quality control. In skeletal muscle, it represses hypertrophy; its attenuation increases lean body mass and basal energy metabolism [36].
*Slc25a11*	Mitochondrial malate/oxoglutarate carrier. Exchanges substrates essential for the Krebs cycle and mitochondrial redox status. Variants that increase its expression enhance thermogenesis in brown adipose tissue (BAT), reducing the risk of obesity and type 2 diabetes [37].
*Myh10*	Myosin heavy chain 10. Not a classic metabolic gene, but its role in cytoskeleton dynamics, cell differentiation, and mechanical signaling may influence adipogenesis, glucose transport and adipose function. Embryonic development, vascular remodeling and tissue organization [38].
*Ampd2*	AMP deaminase 2. Deaminates AMP to IMP in purine metabolism. Its inhibition prevents hepatic triglyceride accumulation induced by high-fructose or high-fat diets, mitigating body weight gain and steatosis [39].
*Gapdhs*	Spermatogenic glyceraldehyde-3-phosphate dehydrogenase. Specific glycolytic enzyme; short isoforms act as a metabolic switch balancing cytosolic glycolysis and mitochondrial oxidative phosphorylation, modifying systemic patterns of carbohydrate storage and oxidation [40].
*Rpl18a*	Ribosomal protein L18a, a component of the 60S subunit. Network analyses of transcriptomic and proteomic data place it within the inflammatory and metabolic response modules activated in adipose tissue during obesity [41,42].
*Tprg1l*	p63-regulated protein type 1 (Mover). Localized at synaptic terminals and associated with the regulation of lipid droplets. Modulates the accumulation and internalization of fatty acids (e.g., oleic acid), protecting cells from lipotoxicity during rapid fat mass gain [43].
*Hcn1*	Hyperpolarization-activated, cyclic nucleotide-gated channel 1. Modulates pacemaker activity and excitability of hypothalamic neurons, regulating firing in circuits that respond to peripheral satiety signals (e.g., leptin) and thereby affecting caloric balance [44].
*Eci2*	Enoyl-CoA delta isomerase 2. Mitochondrial/peroxisomal enzyme required for the efficient breakdown of unsaturated fatty acids; also linked to glucose metabolism [45].

## Data Availability

The mass spectrometry proteomics data have been deposited in the MassIVE repository (Center for Computational Mass Spectrometry, University of California, San Diego) under accession number MSV000103388. Other data supporting the conclusions of this article are available from the corresponding author upon reasonable request.

## Appendix A

**Table A1:** Exploratory candidate proteins with significantly different abundance between Q1 and control rats (Tukey-adjusted *p* ≤ 0.05; not corrected for protein-level multiple testing). Green protein names indicate lower, and red indicate higher, relative abundance in Q1 compared to control rats.

Gene	Uniprot	Fold Change Relative Abundance Q1/Control	*p* Value	Relative Abundance Q1	Relative Abundance Control
*Atp2b3v*	Q64568	0.8940	0.0317	0.0953	0.1066
*Myh10*	Q9JLT0	0.8938	0.0013	0.2092	0.2340
*Tmx2*	Q5XIK2	0.8880	0.0496	0.0534	0.0602
*Tomm70*	Q75Q39	0.8851	0.0398	0.0732	0.0827
*Atp5pd*	P31399	0.8707	0.0377	0.2007	0.2305
*Vsnl1*	P62762	0.8672	0.0127	0.1146	0.1321
*Msn*	O35763	0.8559	0.0071	0.0915	0.1069
*Ndufb1*	P0DN35	0.8478	0.0283	0.0120	0.0142
*Matr3*	P43244	0.8438	0.0061	0.0391	0.0464
*Septin11*	B3GNI6	0.8431	0.0415	0.0745	0.0884
*Sfxn5*	Q8CFD0	0.8414	0.0087	0.0419	0.0498
*Slc25a18*	Q505J6	0.8358	0.0020	0.0530	0.0634
*Atp4a*	P09626	0.8262	0.0367	0.0441	0.0534
*Scn9a*	O08562	0.8255	0.0025	0.0972	0.1177
*Timm9*	Q9WV97	0.8238	0.0450	0.0145	0.0176
*Pgrmc1*	P70580	0.8215	0.0025	0.0480	0.0584
*Rac1*	Q6RUV5	0.8191	0.0191	0.0782	0.0954
*Septin5*	Q9JJM9	0.8174	0.0327	0.0347	0.0425
*Ripor2*	Q7TP54	0.8137	0.0062	0.1142	0.1403
*Atp6v1c1*	Q5FVI6	0.8134	0.0102	0.1049	0.1290
*Capn2*	Q07009	0.8092	0.0139	0.0122	0.0151
*Ppp2r2a*	P36876	0.8089	0.0104	0.0709	0.0876
*Rpl7*	P05426	0.7986	0.0215	0.0158	0.0198
*Nsfl1c*	O35987	0.7974	0.0219	0.0193	0.0242
*Rtn3*	Q6RJR6	0.7955	0.0008	0.1344	0.1689
*Pitpnb*	P53812	0.7929	0.0303	0.0109	0.0137
*Zwint*	Q8VIL3	0.7926	0.0302	0.0237	0.0298
*Gsto1*	Q9Z339	0.7917	0.0015	0.0706	0.0892
*Mogs*	O88941	0.7882	0.0060	0.0290	0.0368
*Cox5a*	P11240	0.7840	0.0415	0.2010	0.2564
*Fth1*	P19132	0.7839	0.0323	0.0116	0.0147
*Ndufs4*	Q5XIF3	0.7794	0.0020	0.0561	0.0720
*Map6*	Q63560	0.7794	0.0222	0.2624	0.3366
*Uqcrq*	Q7TQ16	0.7736	0.0341	0.0335	0.0434
*Camk2d*	P15791	0.7711	0.0372	0.0054	0.0070
*Ran*	P62828	0.7689	0.0241	0.0088	0.0114
*Snap91*	Q05140	0.7683	0.0058	0.1258	0.1637
*Ahcyl1*	B5DFN2	0.7674	0.0122	0.0680	0.0886
*Krt24*	Q6IFX1	0.7640	0.0204	0.0303	0.0396
*Tollip*	A2RUW1	0.7640	0.0344	0.0254	0.0333
*Rpl13a*	P35427	0.7633	0.0048	0.0168	0.0219
*Nrxn1*	Q63372	0.7584	0.0310	0.0321	0.0423
*Prpsap1*	Q63468	0.7578	0.0072	0.0668	0.0881
*Pdgfrb*	Q05030	0.7508	0.0232	0.0650	0.0866
*Cadps*	Q62717	0.7485	0.0445	0.1700	0.2271
*Pkp2*	F1M7L9	0.7481	0.0042	0.0427	0.0570
*Sgta*	O70593	0.7463	0.0146	0.0525	0.0704
*Rhoa*	P61589	0.7424	0.0247	0.0169	0.0227
*Ttr*	P02767	0.7407	0.0374	0.0464	0.0627
*Adgrg1*	Q8K3V3	0.7355	0.0345	0.0282	0.0383
*Acsf2*	Q499N5	0.7327	0.0073	0.0158	0.0215
*Rps10*	P63326	0.7247	0.0010	0.0350	0.0482
*Tmem30a*	Q6AY41	0.7213	0.0022	0.0140	0.0195
*Tppp3*	Q5PPN5	0.7212	0.0026	0.0278	0.0385
*Ces1e*	Q63108	0.7211	0.0171	0.0074	0.0102
*Myh11*	Q63862	0.7187	0.0124	0.0793	0.1103
*Hadha*	Q64428	0.7137	0.0017	0.1212	0.1698
*Stx12*	G3V7P1	0.7109	0.0134	0.0130	0.0183
*Rps9*	P29314	0.7104	0.0196	0.0057	0.0081
*Calb1*	P07171	0.7089	0.0288	0.0967	0.1364
*Wasf1*	Q5BJU7	0.7080	0.0286	0.0121	0.0170
*Lamp1*	P14562	0.7026	0.0009	0.0226	0.0321
*Rpl18a*	P62718	0.7010	0.0001	0.0441	0.0629
*Septin4*	A0A096MJN4	0.6936	0.0474	0.0807	0.1164
*Slc4a8*	Q6RVG2	0.6934	0.0179	0.0145	0.0210
*Psmd13*	B0BN93	0.6919	0.0039	0.0078	0.0113
*Grpel1*	P97576	0.6919	0.0450	0.0121	0.0176
*Pnpla8*	D3ZRC4	0.6913	0.0073	0.0279	0.0404
*Ptprn*	Q63259	0.6900	0.0455	0.0649	0.0941
*Cldn11*	Q99P82	0.6863	0.0138	0.0077	0.0112
*Jam3*	Q68FQ2	0.6830	0.0172	0.0240	0.0352
*Prrt2*	D3ZFB6	0.6734	0.0274	0.0030	0.0044
*Cend1*	Q5FVI4	0.6689	0.0461	0.0270	0.0403
*Emc8*	Q5FVL2	0.6667	0.0032	0.0127	0.0190
*Scp2*	P11915	0.6664	0.0237	0.0832	0.1248
*Cfi*	Q9WUW3	0.6622	0.0026	0.0059	0.0089
*Ascc3*	F1LPQ2	0.6608	0.0123	0.1347	0.2038
*Map1s*	P0C5W1	0.6462	0.0118	0.1021	0.1581
*H1-6*	P06349	0.6385	0.0250	0.0284	0.0444
*Tmem109*	Q6AYQ4	0.6267	0.0035	0.0051	0.0082
*Gsta6*	Q6AXY0	0.6221	0.0361	0.0032	0.0051
*Cops8*	Q6P4Z9	0.6198	0.0341	0.0321	0.0519
*Phyhipl*	Q6AYN4	0.6148	0.0492	0.0455	0.0740
*Tspan2*	Q9JJW1	0.6137	0.0245	0.0028	0.0046
*Ak4*	Q9WUS0	0.5882	0.0042	0.0050	0.0084
*Cul5*	Q9JJ31	0.5600	0.0359	0.0746	0.1331
*Ywhab*	P35213	0.5288	0.0027	0.0372	0.0704
*Sec22b*	Q4KM74	0.4704	0.0071	0.0095	0.0202
*Krt72*	Q6IG04	0.4570	0.0113	0.0088	0.0193
	P11517	0.4276	0.0144	0.4227	0.9885
*Arfgap1*	Q62848	0.4144	0.0453	0.0327	0.0790
*Hba1*	P01946	0.4007	0.0196	0.2626	0.6554
*Rhoq*	Q9JJL4	0.3678	0.0375	0.0118	0.0321
*Hbb*	P02091	0.3134	0.0361	0.0631	0.2013
*Ptges3*	P83868	3.1205	0.0459	0.0224	0.0072
*Apoa1*	P04639	2.8670	0.0210	0.0674	0.0235
*Aqp4*	P47863	2.8636	0.0216	0.0110	0.0038
*Serpina3n*	P09006	2.8171	0.0013	0.0121	0.0043
*C3*	P01026	2.7954	0.0500	0.3206	0.1147
*Ubqln1*	Q9JJP9	2.7188	0.0475	0.0033	0.0012
*Prps2*	P09330	2.3958	0.0183	0.0197	0.0082
*Gapdhs*	Q9ESV6	2.3624	<0.0000	0.0089	0.0037
*Prkaca*	P27791	2.2961	0.0008	0.0022	0.0010
*Cpne1*	D4A1R8	2.2210	0.0084	0.0086	0.0039
*Acsbg1*	Q924N5	2.1779	0.0073	0.0294	0.0135
*Actr2*	Q5M7U6	2.0518	0.0062	0.0087	0.0043
*Afm*	P36953	2.0332	0.0002	0.0849	0.0418
*Pgm1*	P38652	1.9954	<0.0000	0.0188	0.0094
*S1pr1*	P48303	1.9664	0.0004	0.0041	0.0021
*Gpx3*	P23764	1.8775	0.0068	0.0103	0.0055
*Ces1c*	P10959	1.8389	0.0183	0.0192	0.0105
*Plscr3*	Q6QBQ4	1.8303	0.0048	0.0080	0.0044
*Mt-nd4l*	P05507	1.8234	0.0162	0.0024	0.0013
*Prdx4*	Q9Z0V5	1.7891	0.0040	0.0053	0.0029
*Atp6ap2*	Q6AXS4	1.7713	0.0010	0.0096	0.0054
*Timm22*	Q9JKW1	1.7491	0.0017	0.0040	0.0023
*Krt1*	Q6IMF3	1.7333	0.0001	0.1062	0.0613
*Gria2*	P19491	1.7105	0.0301	0.0187	0.0109
*Gad1*	P18088	1.6968	0.0359	0.0932	0.0549
*Gcsh*	Q5I0P2	1.6733	0.0193	0.0025	0.0015
*Actn4*	Q9QXQ0	1.6711	0.0024	0.1066	0.0638
*Ntm*	Q62718	1.6677	0.0123	0.0162	0.0097
*Agt*	P01015	1.6617	0.0437	0.0130	0.0078
*Gpm6b*	Q9JJK1	1.6590	0.0001	0.0129	0.0078
*Ttc17*	B5DEL3	1.6335	0.0126	0.3015	0.1846
*Pafah1b2*	O35264	1.6125	0.0037	0.0060	0.0037
*Prkar2a*	P12368	1.5798	0.0007	0.0092	0.0058
*Cadm3*	Q1WIM3	1.5697	0.0002	0.0174	0.0111
*Cacna2d1*	P54290	1.5661	0.0052	0.1350	0.0862
*Cct2*	Q5XIM9	1.5507	0.0371	0.0362	0.0234
*Ampd2*	Q02356	1.5486	0.0002	0.0398	0.0257
*Macrod1*	Q8K4G6	1.5461	0.0118	0.0075	0.0049
*Hcn1*	Q9JKB0	1.5410	0.0057	0.0289	0.0187
*Sv2c*	Q9Z2I6	1.5218	0.0105	0.0088	0.0058
*Tprg1l*	A8WCF8	1.4991	0.0003	0.0381	0.0254
*Pfn1*	P62963	1.4829	0.0163	0.0063	0.0042
*Cacnb1*	P54283	1.4818	0.0185	0.0052	0.0035
*Cp*	P13635	1.4784	0.0306	0.0351	0.0238
*Arhgdia*	Q5XI73	1.4703	0.0163	0.0176	0.0120
*Ptprs*	Q64605	1.4656	0.0089	0.0503	0.0343
*Cr1l*	Q63135	1.4537	0.0237	0.0188	0.0129
*Adh5*	P12711	1.4520	0.0046	0.1488	0.1025
*Nap1l5*	Q5PPG6	1.4395	0.0293	0.0024	0.0017
*Ppm1e*	Q80Z30	1.4370	0.0201	0.0578	0.0403
*Acat2*	Q5XI22	1.4357	0.0384	0.0175	0.0122
*Slc1a3*	P24942	1.4270	0.0010	0.2346	0.1644
*Mt-cyb*	P00159	1.4157	0.0372	0.0058	0.0041
*Txn*	P11232	1.4132	0.0156	0.0123	0.0087
*Gna13*	Q6Q7Y5	1.4010	0.0091	0.0151	0.0108
*Cap2*	P52481	1.4001	0.0011	0.0168	0.0120
*Dnm3*	Q08877	1.3967	0.0333	0.0419	0.0300
*Uqcrh*	Q5M9I5	1.3942	0.0471	0.0241	0.0173
*Ubr4*	Q2TL32	1.3897	0.0001	0.1473	0.1060
*Serping1*	Q6P734	1.3876	0.0261	0.0159	0.0114
*Gstk1*	P24473	1.3865	0.0077	0.0101	0.0073
*Krt18*	Q5BJY9	1.3852	0.0454	0.1173	0.0847
*Coq9*	Q68FT1	1.3767	0.0314	0.0226	0.0164
*Hsdl2*	Q4V8F9	1.3650	0.0202	0.0049	0.0036
*Ndrg3*	Q6AYR2	1.3581	0.0172	0.0120	0.0089
*Nap1l4*	Q5U2Z3	1.3382	0.0025	0.0295	0.0220
*Fxyd6*	Q91XV6	1.3354	0.0300	0.0310	0.0232
*Xpo1*	Q80U96	1.3341	0.0108	0.0414	0.0310
*Asah1*	Q6P7S1	1.3280	0.0135	0.0212	0.0159
*Slc25a11*	P97700	1.3090	0.0002	0.1413	0.1079
*Pfdn2*	B0BN18	1.3026	0.0082	0.0098	0.0075
*Lonp1*	Q924S5	1.2975	0.0310	0.0345	0.0266
*Gsn*	Q68FP1	1.2942	0.0005	0.0700	0.0541
*Col1a2*	P02466	1.2686	0.0052	0.0995	0.0784
*Ociad1*	Q5XIG4	1.2638	0.0186	0.0313	0.0247
*Hpcal1*	P62749	1.2624	0.0216	0.0886	0.0702
*Uggt1*	Q9JLA3	1.2587	0.0028	0.0706	0.0561
*Hspa8*	P63018	1.2557	0.0085	0.2835	0.2257
*Ap2b1*	P62944	1.2545	0.0003	0.1605	0.1280
*Asrgl1*	Q8VI04	1.1998	0.0386	0.0216	0.0180
*Dpp10*	Q6Q629	1.1920	0.0021	0.0341	0.0286
*Kif15*	Q7TSP2	1.1764	0.0186	0.1487	0.1264
*Stx1b*	P61265	1.1750	0.0070	0.1907	0.1623
*Itih3*	Q63416	1.1616	0.0127	0.0269	0.0232
*Ndufa9*	Q5BK63	1.1433	0.0416	0.0755	0.0660
*Ryr2*	B0LPN4	1.1320	0.0309	0.1632	0.1442
*Slc25a12*	F1LX07	1.1249	0.0365	0.2612	0.2322

**Table A2:** Exploratory candidate proteins with significantly different abundance between Q2 and control rats (Tukey-adjusted *p* ≤ 0.05; not corrected for protein-level multiple testing). Green protein names indicate lower, and red indicate higher, relative abundance in Q2 compared to control rats.

Gene	Uniprot	Fold Change Relative Abundance Q2/Control	*p* Value	Relative Abundance Q2	Relative Abundance Control
*Ripor2*	Q7TP54	0.8511	0.0311	0.1194	0.1403
*Eef1g*	Q68FR6	0.8426	0.0216	0.0265	0.0314
*Aldh1l1*	P28037	0.8361	0.0498	0.0252	0.0302
*Camkv*	Q63092	0.8331	0.0448	0.0162	0.0195
*Dctn1*	P28023	0.8282	0.0150	0.0476	0.0574
*Prpsap1*	Q63468	0.8126	0.0437	0.0716	0.0881
*Lamp1*	P14562	0.8021	0.0342	0.0258	0.0321
*Pkp2*	F1M7L9	0.7921	0.0179	0.0452	0.0570
*Tppp3*	Q5PPN5	0.7595	0.0081	0.0292	0.0385
*Ncl*	P13383	0.7396	0.0434	0.0591	0.0799
*Sh3gl3*	O35180	0.6884	0.0175	0.0153	0.0223
*Trim23*	P36407	0.6835	0.0382	0.0528	0.0773
*Ywhab*	P35213	0.6281	0.0183	0.0442	0.0704
*Gsta6*	Q6AXY0	0.6149	0.0235	0.0031	0.0051
*Sh3glb2*	Q5PPJ9	0.5709	0.0373	0.0245	0.0428
*Sec22b*	Q4KM74	0.4671	0.0045	0.0094	0.0202
*Serpind1*	Q64268	2.4537	0.0367	0.0347	0.0141
*Acsbg1*	Q924N5	2.0326	0.0171	0.0275	0.0135
*Prkaca*	P27791	1.9035	0.0228	0.0019	0.0010
*Actr2*	Q5M7U6	1.8397	0.0312	0.0078	0.0043
*Afm*	P36953	1.8211	0.0027	0.0761	0.0418
*Rps7*	P62083	1.7187	0.0099	0.0016	0.0010
*Gad1*	P18088	1.6848	0.0308	0.0925	0.0549
*Timm22*	Q9JKW1	1.6576	0.0048	0.0038	0.0023
*Ntm*	Q62718	1.6380	0.0131	0.0159	0.0097
*Pgm1*	P38652	1.6087	0.0131	0.0151	0.0094
*Macrod1*	Q8K4G6	1.6048	0.0029	0.0078	0.0049
*Krt1*	Q6IMF3	1.5623	0.0025	0.0957	0.0613
*Actn4*	Q9QXQ0	1.5572	0.0108	0.0993	0.0638
*Scarb2*	P27615	1.4664	0.0082	0.0250	0.0171
*Hspa2*	P14659	1.4282	0.0467	0.0190	0.0133
*Uqcrh*	Q5M9I5	1.3970	0.0344	0.0241	0.0173
*Gpm6b*	Q9JJK1	1.3860	0.0193	0.0108	0.0078
*Ndufa11*	Q80W89	1.3537	0.0359	0.0160	0.0118
*Cadm3*	Q1WIM3	1.3495	0.0272	0.0150	0.0111
*Slc1a3*	P24942	1.3446	0.0072	0.2211	0.1644
*Hnrnpa3*	Q6URK4	1.3442	0.0338	0.0079	0.0058
*Pip4k2b*	O88377	1.3260	0.0402	0.0285	0.0215
*Eci2*	Q5XIC0	1.3181	0.0002	0.0068	0.0052
*M6pr*	Q6AY20	1.2986	0.0129	0.0056	0.0043
*Cap2*	P52481	1.2925	0.0188	0.0156	0.0120
*Iqsec3*	Q76M68	1.2734	0.0131	0.0400	0.0314
*Pcyox1*	Q99ML5	1.2712	0.0361	0.0225	0.0177
*Psmd11*	F1LMZ8	1.2506	0.0464	0.0143	0.0114
*Ndufa10*	Q561S0	1.2087	0.0212	0.0837	0.0692
*Pitpna*	P16446	1.2081	0.0460	0.0109	0.0090
*Prxl2a*	Q6AXX6	1.2004	0.0404	0.0136	0.0113
*Mtdh*	Q9Z1W6	1.1551	0.0495	0.0378	0.0327
*Entpd2*	O35795	1.1493	0.0475	0.0230	0.0200
*Ndufs2*	Q641Y2	1.1418	0.0153	0.1039	0.0910
*Folh1*	P70627	1.1318	0.0261	0.0976	0.0863
*Slc6a9*	P28572	1.1222	0.0209	0.0380	0.0339

**Table A3:** Exploratory candidate proteins with significantly different abundance between Q3 and control rats (Tukey-adjusted *p* ≤ 0.05; not corrected for protein-level multiple testing). Green protein names indicate lower, and red indicate higher, relative abundance in Q3 compared to control rats.

Gene	Uniprot	Fold Change Relative Abundance Q3/Control	*p* Value	Relative Abundance Q3	Relative Abundance Control
*Dctn1*	P28023	0.8441	0.0428	0.0485	0.0574
*Matr3*	P43244	0.8414	0.0052	0.0390	0.0464
*C4bpa*	Q63514	0.8320	0.0429	0.0490	0.0588
*Aldh3a2*	P30839	0.8141	0.0336	0.0362	0.0444
*Hadha*	Q64428	0.7950	0.0381	0.1350	0.1698
*Pitpnb*	P53812	0.7888	0.0261	0.0108	0.0137
*Maob*	P19643	0.7866	0.0286	0.0949	0.1206
*Psmd13*	B0BN93	0.7698	0.0476	0.0087	0.0113
*Pkp2*	F1M7L9	0.7628	0.0077	0.0435	0.0570
*Mt-atp8*	P11608	0.7545	0.0371	0.0367	0.0487
*Tppp3*	Q5PPN5	0.7543	0.0094	0.0290	0.0385
*Ces1e*	Q63108	0.7491	0.0388	0.0077	0.0102
*Ncl*	P13383	0.7225	0.0364	0.0578	0.0799
*Prrt2*	D3ZFB6	0.6796	0.0317	0.0030	0.0044
*Ywhab*	P35213	0.5886	0.0106	0.0414	0.0704
*Sec22b*	Q4KM74	0.5783	0.0460	0.0117	0.0202
*Actr2*	Q5M7U6	2.1203	0.0032	0.0090	0.0043
*Afm*	P36953	1.8563	0.0026	0.0776	0.0418
*Pgm1*	P38652	1.8260	0.0007	0.0172	0.0094
*Mt-nd4l*	P05507	1.7706	0.0275	0.0023	0.0013
*Cotl1*	B0BNA5	1.7123	0.0025	0.0026	0.0015
*Prdx4*	Q9Z0V5	1.6656	0.0197	0.0049	0.0029
*Rplp1*	P19944	1.6454	0.0457	0.0048	0.0029
*Pafah1b2*	O35264	1.6298	0.0027	0.0061	0.0037
*Ttc17*	B5DEL3	1.6215	0.0149	0.2993	0.1846
*Pfn1*	P62963	1.6045	0.0017	0.0068	0.0042
*Timm22*	Q9JKW1	1.5940	0.0177	0.0037	0.0023
*Scarb2*	P27615	1.5913	0.0009	0.0272	0.0171
*Atp2a1*	Q64578	1.5744	0.0139	0.0147	0.0094
*Pebp1*	P31044	1.5681	0.0331	0.0443	0.0283
*Ddt*	P80254	1.5602	0.0152	0.0104	0.0067
*Actn4*	Q9QXQ0	1.5402	0.0197	0.0983	0.0638
*Krt1*	Q6IMF3	1.5316	0.0068	0.0939	0.0613
*Aldh1a7*	P13601	1.5242	0.0088	0.0034	0.0022
*Prkar2a*	P12368	1.4701	0.0074	0.0085	0.0058
*Rpsa*	P38983	1.4686	0.0004	0.0093	0.0064
*Hspa2*	P14659	1.4526	0.0415	0.0193	0.0133
*Adcy5*	Q04400	1.4237	0.0285	0.0751	0.0528
*Oxct1*	B2GV06	1.4183	0.0047	0.0192	0.0135
*Ndufa11*	Q80W89	1.4123	0.0144	0.0167	0.0118
*Stx1a*	P32851	1.4112	0.0007	0.0445	0.0315
*Gpm6b*	Q9JJK1	1.4047	0.0179	0.0110	0.0078
*Coq9*	Q68FT1	1.3986	0.0202	0.0230	0.0164
*Ptprs*	Q64605	1.3886	0.0394	0.0476	0.0343
*Serping1*	Q6P734	1.3813	0.0296	0.0158	0.0114
*Sec11c*	Q9WTR7	1.3490	0.0115	0.0050	0.0037
*Ppia*	P10111	1.3327	0.0081	0.1090	0.0818
*Dync1i2*	Q62871	1.3324	0.0486	0.0458	0.0344
*Slc22a17*	Q9P290	1.3323	0.0344	0.0051	0.0038
*Gdi2*	P50399	1.3322	0.0273	0.0133	0.0100
*Psmd11*	F1LMZ8	1.3247	0.0077	0.0151	0.0114
*Cap2*	P52481	1.3203	0.0119	0.0159	0.0120
*Eno2*	P07323	1.3179	0.0135	0.1733	0.1315
*Pcyox1*	Q99ML5	1.3092	0.0176	0.0232	0.0177
*Tpi1*	P48500	1.3015	0.0331	0.1263	0.0971
*Ctbp1*	Q9Z2F5	1.2950	0.0396	0.0162	0.0125
*Bin1*	O08839	1.2927	0.0479	0.0500	0.0387
*Vgf*	P20156	1.2675	0.0309	0.0287	0.0227
*Tst*	P24329	1.2626	0.0454	0.0429	0.0340
*Iqsec3*	Q76M68	1.2481	0.0384	0.0392	0.0314
*Prkcb*	P68403	1.2458	0.0320	0.0272	0.0218
*Erp29*	P52555	1.2406	0.0275	0.1065	0.0859
*Abcb8*	Q5RKI8	1.2394	0.0471	0.0263	0.0212
*Camsap1*	D3Z8E6	1.2356	0.0296	0.0446	0.0361
*Gripap1*	Q9JHZ4	1.2331	0.0457	0.0813	0.0659
*Ndufa10*	Q561S0	1.2312	0.0122	0.0853	0.0692
*Pitpna*	P16446	1.2309	0.0288	0.0111	0.0090
*Atp1b3*	Q63377	1.2278	0.0329	0.0497	0.0405
*Idh3g*	P41565	1.2251	0.0342	0.0359	0.0293
*Calb2*	P47728	1.2191	0.0092	0.0496	0.0407
*Slc25a4*	Q05962	1.2152	0.0301	0.4799	0.3949
*Septin7*	Q9WVC0	1.2141	0.0380	0.0677	0.0557
*Prxl2a*	Q6AXX6	1.2138	0.0334	0.0137	0.0113
*Eci2*	Q5XIC0	1.2111	0.0267	0.0063	0.0052
*Sirt2*	Q5RJQ4	1.2072	0.0187	0.1313	0.1088
*Mtdh*	Q9Z1W6	1.2037	0.0074	0.0394	0.0327
*Dync1h1*	P38650	1.1703	0.0038	0.2561	0.2189
*Dmd*	P11530	1.1581	0.0200	0.1873	0.1617
*Dnah7*	Q63170	1.1420	0.0476	0.1650	0.1444

**Table A4:** Exploratory candidate proteins with significantly different abundance between Q4 and control rats (Tukey-adjusted *p* ≤ 0.05; not corrected for protein-level multiple testing). Red protein names indicate higher relative abundance in Q4 compared to control rats.

Gene	Uniprot	Fold Change Relative Abundance Q4/Control	*p* Value	Relative Abundance Q4	Relative Abundance Control
*S1pr1*	P48303	1.8271	0.0026	0.0038	0.0021
*Armc10*	B1WBW4	1.7690	0.0014	0.0015	0.0009
*Capza1*	B2GUZ5	1.5719	0.0124	0.0078	0.0050
*Pafah1b2*	O35264	1.5342	0.0138	0.0057	0.0037
*Kcna4*	P15385	1.4716	0.0206	0.0109	0.0074
*Rps8*	P62243	1.3660	0.0374	0.0055	0.0040
*Ywhag*	P61983	1.3014	0.0431	0.0517	0.0397
*Nap1l4*	Q5U2Z3	1.2574	0.0316	0.0277	0.0220

**Table A5:** Proteins comprising the lower-weight-gain-associated profile (n = 44), defined as the overlap between the Q1 vs. control comparison (this study; Tukey-adjusted *p* ≤ 0.05) and the Q1 vs. Q4 comparison (previous study [12]) (Figure 5A). Proteins in bold were also FDR-supported (Table 1). This profile is exploratory and requires independent validation.

Gene	Protein	Fold Change (Q1/Control)	*p* (Tukey)
*Apoa1*	Apolipoprotein A-I	2.87	0.0210
*Serpina3n*	Serine protease inhibitor A3N	2.82	0.0013
*Prps2*	Ribose-phosphate pyrophosphokinase 2	2.40	0.0183
***Gapdhs***	**Glyceraldehyde-3-phosphate dehydrogenase, testis-specific**	**2.36**	**<0.0001**
*Acsbg1*	Long-chain-fatty-acid--CoA ligase ACSBG1	2.18	0.0073
***Pgm1***	**Phosphoglucomutase-1**	**2.00**	**<0.0001**
*Gpx3*	Glutathione peroxidase 3	1.88	0.0068
*Ces1c*	Carboxylesterase 1C	1.84	0.0183
*Plscr3*	Phospholipid scramblase 3	1.83	0.0048
*Atp6ap2*	Renin receptor	1.77	0.0010
***Gpm6b***	**Neuronal membrane glycoprotein M6-b**	**1.66**	**0.0001**
***Cadm3***	**Cell adhesion molecule 3**	**1.57**	**0.0002**
*Cacna2d1*	Voltage-dependent calcium channel subunit alpha-2/delta-1	1.57	0.0052
***Ampd2***	**AMP deaminase 2**	**1.55**	**0.0002**
***Hcn1***	**Potassium/sodium hyperpolarization-activated cyclic nucleotide-gated channel 1**	**1.54**	**0.0057**
*Sv2c*	Synaptic vesicle glycoprotein 2C	1.52	0.0105
***Tprg1l***	**Tumor protein p63-regulated gene 1-like protein**	**1.50**	**0.0003**
*Arhgdia*	Rho GDP-dissociation inhibitor 1	1.47	0.0163
***Ubr4***	**E3 ubiquitin-protein ligase UBR4**	**1.39**	**0.0001**
*Gstk1*	Glutathione S-transferase kappa 1	1.39	0.0077
***Slc25a11***	**Mitochondrial 2-oxoglutarate/malate carrier protein**	**1.31**	**0.0002**
*Uggt1*	UDP-glucose:glycoprotein glucosyltransferase 1	1.26	0.0028
*Kif15*	Kinesin-like protein KIF15	1.18	0.0186
*Stx1b*	Syntaxin-1B	1.18	0.0070
*Tmem109*	Voltage-gated monoatomic cation channel TMEM109	0.63	0.0035
*Cfi*	Complement factor I	0.66	0.0026
*Emc8*	ER membrane protein complex subunit 8	0.67	0.0032
*Psmd13*	26S proteasome non-ATPase regulatory subunit 13	0.69	0.0039
***Rpl18a***	**Large ribosomal subunit protein eL20**	**0.70**	**0.0001**
*Wasf1*	Actin-binding protein WASF1	0.71	0.0286
***Prpsap1***	**Phosphoribosyl pyrophosphate synthase-associated protein 1**	**0.76**	**0.0072**
*Rpl13a*	Large ribosomal subunit protein uL13	0.76	0.0048
*Ran*	GTP-binding nuclear protein Ran	0.77	0.0241
*Camk2d*	Calcium/calmodulin-dependent protein kinase type II subunit delta	0.77	0.0372
*Fth1*	Ferritin heavy chain	0.78	0.0323
*Gsto1*	Glutathione S-transferase omega-1	0.79	0.0015
*Rtn3*	Reticulon-3	0.80	0.0008
*Pgrmc1*	Membrane-associated progesterone receptor component 1	0.82	0.0025
*Sfxn5*	Sideroflexin-5	0.84	0.0087
*Septin11*	Septin-11	0.84	0.0415
*Ndufb1*	NADH dehydrogenase [ubiquinone] 1 beta subcomplex subunit 1	0.85	0.0283
*Vsnl1*	Visinin-like protein 1	0.87	0.0127
*Tomm70*	Mitochondrial import receptor subunit TOM70	0.89	0.0398
***Myh10***	**Myosin-10**	**0.89**	**0.0013**
